# The Impacts of Pregnancy on Cognition and Cell Proliferation in a Live‐Bearing Fish ( *Poeciliopsis gracilis* )

**DOI:** 10.1111/ejn.70523

**Published:** 2026-05-08

**Authors:** Tiffany R. Ernst, Alianne Keijzer, Sofia Vellere, Anthony Lee, Johan L. van Leeuwen, Alexander Kotrschal, Aniko Korosi, Bart J. A. Pollux

**Affiliations:** ^1^ Wageningen University, Department of Animal Sciences Experimental Zoology Group Wageningen the Netherlands; ^2^ School of Advanced Studies, Center for Neuroscience, Pharmacology Unit University of Camerino Camerino Italy; ^3^ Swammerdam Institute of Life Sciences, Center for Neuroscience, Brain Plasticity Group University of Amsterdam Amsterdam the Netherlands; ^4^ Department of Animal Sciences, Behavioral Ecology Group Wageningen University Wageningen the Netherlands

**Keywords:** cognitive flexibility, Poeciliidae, spatial learning

## Abstract

Pregnancy is a high‐energy process which temporarily decreases cognitive function and affects the neurogenic capacity of the maternal brain. Pregnant females exhibit decreased performance in spatial memory tasks which has been linked to altered neurogenesis in the dentate gyrus of the hippocampus. While these processes are well‐resolved for mammals, whether they are conserved across other, nonmammalian, live‐bearing animal lineages without placentation remains enigmatic. Here, we test the relationship between pregnancy and cognition in the live‐bearing fish *Poecilipsis gracilis*. Female *P. gracilis* are almost continuously pregnant after sexual maturation meaning that any cognitive deficits due to pregnancy may be constant throughout adulthood. To determine the consequences of this continuous pregnancy on maternal cognition, we compared the performance of pregnant and virgin females in two ecologically relevant cognitive assays, a spatial memory task and a reversal learning task. To further assess pregnancy‐induced changes in brain plasticity, the brains of each female were then assessed using immunohistochemical staining for the neurogenic proliferation marker *ki67*. We found that pregnant females showed a decline in spatial learning performance, exhibiting more non‐choice trials. Although pregnant females did not exhibit decreased cell proliferation in the hippocampal‐analogous region of the brain, they did show decreased proliferation in the olfactory bulb and ventral telencephalon. Our results indicate that, just like in mammals, pregnancy in fish impacts female cognitive capacity and cell proliferation, even though those poeciliid fishes do not have a placenta.

## Graphical abstract

We assessed the impact of pregnancy on cognition and cell proliferation in the live‐bearing fish *Poeciliopsis gracilis* . Pregnant females showed reduced spatial learning but unchanged reversal learning, alongside decreased cell proliferation in the olfactory bulb and ventral telencephalon, indicating pregnancy‐induced cognitive and neural changes in a nonplacental species.

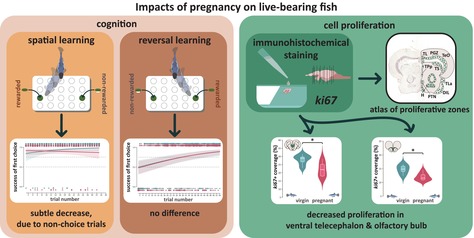

**Abbreviations**
Cce: corpus cerebelli
D: dorsal telencephalon
Dc: dorsocentral telencephalon
DG: dentate gyrus of the hippocampus
Di: dorsolateral telencephalon
Dm: dorsomedial telencephalon
Dp: dorsoposterior telencephalon
H: hypothalamus
ICL: olfactory bulb, internal cellular layer
PGZ: periventricular gray zone of the TeO
SVZ: subventricular zone
TeO: optic tectum
V: ventral telencephalon
Vd: ventral telencephalon, dorsal nucleus
Vp: ventral telencephalon, postcommissural nucleus
Vv: ventral telencephalon, nucleus

## 1 Introduction

Pregnancy is an energetically expensive physiologic process which impacts nearly every part of the maternal body. Throughout gestation, mothers must provide a hospitable environment for their developing embryo(s), facilitating gas exchange across the maternal–fetal interface, preventing immunological rejection of the fetus, and providing nutrients for growth and development. Meanwhile, mothers undergo drastic physiological changes during pregnancy which impact maternal body weight/size/fat composition, metabolism, locomotive performance, behavior, mood, and cognition. While many of these adaptations are necessary for maintaining a healthy pregnancy, these substantial and dynamic changes can also have detrimental impacts on a mother's mood, behavior, and cognition. Pregnant women often report a plethora of cognitive deficits during pregnancy, including decreased memory retention, decreased verbal and spatial memory, and decreased executive functioning, resulting in a phenomenon commonly referred to as “Baby Brain” (Davies et al. 2018; Henry and Rendell 2007; Barda et al. 2021; de Groot et al. 2006).

One form of neural plasticity modulated by pregnancy is adult neurogenesis (AN): the process by which neural stem cells proliferate, differentiate, and mature into new neurons. In mammals, AN occurs primarily in only two regions of the brain, the subventricular zone (SVZ) and the dentate gyrus (DG) of the hippocampus (Zhao et al. 2008). Research in rodent models has found that pregnancy induces an increase in AN in the SVZ, primarily stimulated by elevated levels of prolactin throughout pregnancy (Shingo et al. 2003); neural stem cells produced in this region migrate to the olfactory bulb, improving both olfaction and the olfactory discrimination necessary for mothers to recognize and behaviorally respond to their offspring (Medina and Workman 2020). Conversely, pregnancy either has no effect or a detrimental effect on AN in the DG of the hippocampus which has been associated with decreased spatial memory, particularly during late‐pregnancy and postpartum (Medina and Workman 2020; Galea et al. 2013; Duarte‐Guterman et al. 2015). Given that spatial memory has been intrinsically linked with hippocampal size and function in a variety of species (Sherry et al. 1992) and that pregnant individuals also exhibit decreased hippocampal volumes (humans and rats; Galea et al. (2000); Hoekzema et al. (2017)), research has suggested that decreased spatial memory performance during pregnancy might be associated with hippocampal neurogenesis. Researchers have proposed that decreased spatial memory during pregnancy may be part of the evolutionary trade‐off necessary to both manage maternal energy expenditure in favor of offspring growth and to redirect the maternal brain toward increased emotional processing and offspring care (Ziomkiewicz et al. 2019; Sherry and Hampson 1997).

While pregnancy‐modulated neural plasticity has been extensively researched in mammals, very little work has been directed toward species which have evolved a live‐bearing reproductive strategy outside of the mammalian lineage. Fishes were the first vertebrates to evolve live‐bearing reproduction (Long et al. 2008), making them an interesting model for understanding the evolutionary basis of vertebrate, live‐bearing reproduction (Pollux et al. 2009). Across live‐bearing fish species, there is a spectrum of live‐bearing strategies including placental and nonplacental species (Pollux et al. 2009), which provides a unique opportunity to disentangle the role of pregnancy itself vs. the role of the placenta in shaping maternal neural plasticity. Moreover, fishes exhibit dramatically more neurogenesis in adulthood than mammals, allowing for far greater neural plasticity (Ganz and Brand 2016). While mammalian neurogenesis is restricted to the SVZ and the DG of the hippocampus, teleost fishes have 12–16 identified proliferative zones with proliferation rates nearly 2 magnitudes higher than those in the adult mammalian brain (Ganz and Brand 2016; Zupanc 2011). Studies across a wide range of teleost fishes show that this high neurogenic capacity and the localization of these proliferative zones are highly conserved (Ganz and Brand 2016). Thereby, fishes offer a unique model to understand how neural plasticity via neurogenesis and subsequently maternal cognition may be modulated during pregnancy in nonmammalian live‐bearers without placentation.

With this study, we aim to make the first foray into investigating the impacts of pregnancy on maternal cognition and neurogenesis in a live‐bearing fish ( *Poeciliopsis gracilis* ) from the family Poeciliidae. *P. gracilis* is a particularly interesting model species because it exemplifies several specialized reproductive adaptations: (1) *P. gracilis* is a live‐bearing fish species which lacks a placenta, whereby embryos are retained in the ovary during gestation and receive nutrients from a pre‐provisioned yolk, a reproductive strategy known as lecithotrophic viviparity (Pollux et al. 2014). With this strategy, *P. gracilis* females may experience a lower energetic demand during pregnancy (i.e., between fertilization and giving birth) than placental live‐bearers who have to continuously nourish their offspring throughout gestation. (2) In addition to being live‐bearing, *P. gracilis* females are superfetatious, meaning females are able to gestate more than one brood of offspring simultaneously. While a typical pregnancy lasts ≈ 4 weeks, females become fertile every ≈ 2 weeks, resulting in a near‐continuous pregnancy of temporally overlapping broods. This near‐continuous pregnancy may have differential impacts on pregnancy‐induced neural plasticity than those seen in mammals where pregnancy is a more temporary state. (3) While pregnancy‐induced cognitive impairment in mammals has been hypothetically linked to the energy trade‐offs required to prepare the maternal brain for postnatal maternal care behaviors, *P. gracilis* females exhibit no postnatal maternal care, and it is therefore unclear if or to what extent such a cognitive trade‐off exists in our fishes.

Using this unique model species, our study aims (1) to determine if, and to what extent, maternal cognition is impacted in pregnant, lecithotrophic live‐bearing females, (2) to determine whether this pregnancy impacts cell proliferation in the maternal brain, and (3) to generate an atlas of proliferative zones in the brain of a poeciliid fish. We tested behavioral performance of virgin and pregnant *P. gracilis* in a spatial memory task. Given that high hippocampal neurogenesis in nonpregnant individuals is important for cognitive flexibility (Anacker and Hen 2017), we followed our spatial memory task with a single test of reversal learning. After behavioral testing, we assessed cell proliferation in the maternal brains using immunohistochemical (IHC) staining for the cell proliferation marker *ki67*. By studying maternal cognition and neuronal cell proliferation in *P. gracilis* , we aim to understand whether the evolution of a live‐bearing reproductive strategy necessitates certain maternal neurologic trade‐offs to appropriately adapt for a healthy pregnancy.

## 2 Materials and Methods

**FIGURE 1:**
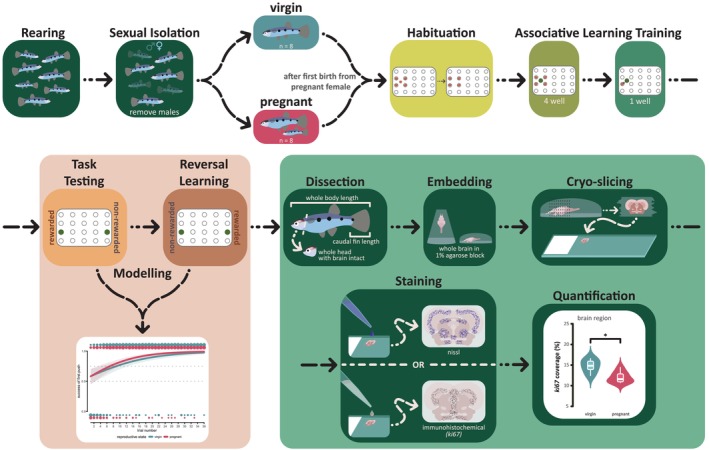
Schematic flow‐chart of the research methodology. Chart is read from left to right following the black arrows. See main text and Appendix S2 for a detailed description.

Figure 1 gives a visual overview of the experimental workflow which is described in detail in the sections below.

### 2.1 Experimental Animals

*Poeciliopsis gracilis* used in this experiment were reared and maintained in the Live‐Bearing Fish Facility of CARUS at Wageningen University and Research (Wageningen,the Netherlands). Fish were housed in tanks with constant aeration and a water temperature of 25°C ± 2°C under a light–dark cycle of 12 h light to 12 h dark. Prior to experiments, fish were fed ad libitum sterilized CAVIAR (BernAqua) and nauplii *Artemia* spp. (Salt Lake Aquafeed, Premium Artemia Cysts, hatched on‐site) daily at 8:00 and 16:00, respectively. Fish received either ad libitum liver paste (Appendix S1) or REPASHY grub pie (Insectivore Gel Premix for Fish) at 12:00 on Mondays, Wednesdays, and Fridays. *P. gracilis* used in this study were used under approval by the animal ethics committee of Wageningen University and Research (2020.W‐0027).

Fish were reared in 30‐L community tanks at a density of 1 fish per liter until sexual maturation. Males were removed from the rearing tanks before reaching sexual maturity to ensure virginity of all the female fish. At ∼3 months of age, female fish were randomly divided into two groups—virgin and pregnant—and rehoused in experimental tanks. Virgin fish were housed individually to maintain virginity, while pregnant individuals were housed with one male to allow for reproduction. Experimental tanks consisted of a 13‐L compartment within a larger six‐compartment tank (Figure S1a,b). This compartment structure allowed fish to see, smell, and visually interact with neighboring fish, reducing isolation stress particularly in virgin females. Each tank contained a plastic aquarium plant for fish to use as shelter and enrichment.

### 2.2 Behavioral Training and Testing

Once a pregnant female had given birth to her first brood of offspring, she was moved to a behavioral tank, and a randomly selected virgin female was moved to a nearby behavioral tank; this pair of females then formed an experimental block. Fish, therefore, began behavior training within 24 h of their first brood. The male from the pregnant female was subsequently relocated to a nearby stock tank. Behavioral tanks were identical in size and shape to the home tanks but were modified to accommodate behavioral testing. The front 13 cm (depth) of each compartment was lined with opaque gray panels to prevent the fish from seeing into neighboring compartments during the behavioral testing and learning from watching their neighbors (Brown and Laland 2003). Each tank was equipped with a pair of guillotine doors, one opaque and one translucent, to separate the home compartment from the behavioral arena (Figure S1c,d). During training and testing, the fish were no longer fed ad libitum and instead only received food during the behavioral trials; this meant that fish were kept on total food restriction outside of the testing periods (including during the weekends) to increase appetite and performance during the testing periods.

All behavioral training and testing was observed and scored by two experimenters simultaneously. Behavioral training consisted of a short period of habituation followed by a longer period of associative learning training, where fish had to learn to associate a food reward with the location of a green disk; fish were allowed to learn at their own pace up until a maximum of ∼30 days. After the fish reached the predetermined learning criteria or met the 30‐day threshold, they proceeded to behavioral testing where they were assessed first in a spatial learning task, followed directly by a reversal learning task. The general protocol for behavioral training and testing was derived from previous work by Ernst et al. (2025) and is described in more detail in Appendix S2.

### 2.3 Dissection and Brain Preparation

Within 24 h after completing their last behavioral trial, fish were sacrificed with an overdose of 2‐phenoxyethanol (≥ 2 ml/L). Additional virgin, female fish from the same rearing cohort which did not undergo behavioral training/testing were similarly sacrificed/dissected/stained to generate an atlas of baseline neural proliferative zones in *P. gracilis* . Fish were then weighed to determine their wet mass, and a Mitutoyo Absolute Digimatic electronic caliber (CD–15CP) was used to measure the total length and caudal fin length, as shown in Figures 1 and S2. These measurements were used to calculate standard length (total length − caudal fin length). To preserve the brain, fish were decapitated using surgical scissors by making a dorsoventral cut just posterior to the operculum (Figure 1). The whole head was then fixed in 4% para‐formaldehyde (PFA) in 1X phosphate‐buffered saline (PBS) for a maximum of 2 weeks. After fixation, the heads were rinsed in tap water and dissected to remove the brain which was stored for a maximum of 3 days in 1X PBS or immediately embedded.

All brains were embedded individually in 1% agarose 5% sucrose in deionized water (DI) and then fully inundated in 30% sucrose prior to flash‐freezing in liquid nitrogen. The brains were oriented in the agarose blocks so that they could be cut coronally, from the anterior to posterior end, as shown in Figure 1. Eighteen‐micrometer‐thick coronal sections were cut with the Cryostat at −20^°^C/−21°C (object/chamber) into a nine‐slide series. Slides were stored at −80°C until ready for staining.

### 2.4 Nissl Staining

To visualize neuronal morphology, one slide from each nine‐slide series underwent standard Nissl staining. Staining was performed at room temperature. Slides were submerged in the cresyl violet acetate solution 0.02%–0.25% (K21988335, Certistain) for 15–20 min and then shortly rinsed in distilled water. Slides were dehydrated following a standard dehydration series (50%, 70%, 96%, and 2 × 100% ethanol for 2 min each, followed by 2 × 5 min xylene) and mounted in DEPEX.

### 2.5 IHC Staining: *ki67*

To assess neuronal cell proliferation, one slide from each nine‐slide series underwent IHC staining for the cell proliferation marker *ki67*. Slides were brought to room temperature and rinsed in 0.1 M PBS (pH 7.4). Endogenous peroxidase activity was blocked in 0.5% H_2_O_2_ in PBS for 15 min. After rinsing again in PBS, slides were pre‐incubated in 2% milk powder +1% Triton in PBS for 30 min. Slides were rinsed in PBS and then incubated in 1:300 primary rabbit polyclonal antibody anti‐*ki67* (Merck Millipore AB9260) in 2% milk power +1% Triton (TRITON X‐100, Sigma Aldrich) in PBS for 1 h at room temperature and then overnight at 4°C. After rinsing in PBS, slides were incubated in the secondary antibody, a 1:200 goat antirabbit biotinylated vector (Vector Laboratories, 6‐BA‐1000) for 2 h at room temperature. Subsequently, the slides were rinsed in PBS and incubated in ABC‐elite (VECTASTAIN Elite ABC‐horseradish peroxidase (HRP), PK‐6100) at 1:800 dilution in PBS for 2 h at room temperature. Slides were washed first in PBS and then three times in 0.05 M Tris‐Buffer (TB, pH 7.6). To give substrate to the HRP, slides were incubated for 11 min in 3‐3′‐diaminobenzidine (DAB) + 0.23% nickel sulfate + 0.04% H_2_O_2_. The reaction was stopped with fast washes in TB and then slides were dehydrated before mounting in DEPEX. Negative controls were included by omitting the primary antibody during the first incubation step.

### 2.6 Imaging and Quantification

All slides were imaged with a Nikon Eclipse upright stage microscope equipped with a Nikon DS‐Ri2 CMOS color camera for wide‐field microscopy. Images were acquired using NIS‐Elements Basic Research software. The following steps were performed for virgin fish which did not undergo behavioral testing to generate the atlas and for both virgin and pregnant fish which were part of the experimental protocol. Prior to quantification, fish were assigned a random number and thereby quantified “blindly” to remove experimenter bias. To determine the neurogenic areas, brain slices stained for *ki67* were matched with their corresponding slice stained with Nissl; the images of these slices were then overlaid and the borders of the neurogenic regions were traced according to the neuronal morphology (Nissl) and the *ki67+* coverage. Brain regions were defined in the atlas by comparing morphological characteristics of our brain slices after either Nissl or *ki67+* staining to established and annotated brain atlases for *Danio rerio* (Wulliman et al. 1996) and *Poecilia reticulata* (Fischer et al. 2018). From these identified neurogenic zones, we decided to quantify *ki67+* coverage in a selection of regions: dorsal telencephalon (homologous to the pallium in mammals and important for various types of learning), hypothalamus (important for hormonal regulation during pregnancy), olfactory bulb (integral for olfaction), ventral telencephalon (homologous to the basal ganglia in mammals), and cerebellum (important for motor function). Image quantification was performed using Fiji2 (Fiji Is Just ImageJ, version 2.3.1) by measuring the percent area covered by *ki67+* cells in each of the aforementioned regions for all virgin and pregnant fish.

### 2.7 Data Analysis

Behavioral data were collected from 15 fish in total: 7 virgin and 8 pregnant fish. One fish (V05) reached a humane endpoint during the 1 well associative learning subphase and therefore did not perform the spatial and reversal learning tasks (Figure S3a). Due to a freezer failure during sample storage, Nissl/IHC staining was only performed on brain slices from 14 fish: 6 virgin (excludes V11) and 8 pregnant fish. All data analyses were performed in R version 4.3.2 (R Core Team 2023) in RStudio (Posit team 2023). All descriptive data and associative learning data (weight, standard length, # total number of training trials; Figures S2 and S3) and IHC *ki67* coverage data were tested for normality using a Shapiro–Wilks Normality test. Data which fit the assumption of Gaussian normality were analyzed using a two‐sided *T*‐test, while nonnormal data were analyzed with a Wilcoxon rank sum test.

All behavioral data were analyzed using the *lme4* package (Bates et al. 2015), and model predictions were plotted using the *effects* package (Fox and Weisberg 2019; Fox 2003). For both the spatial and reversal learning tasks, we compared the performance of virgin and pregnant fish in the following:

- Success of the first disk push (1 = success; 0 = fail), where non‐choice trials were treated as incorrect, using a GLMM (binomial) with reproductive status (virgin or pregnant), trial number in phase, and reproductive status × trial number in phase as predictor variables and fish identity as a random variable.
- Whether fish make a choice (1 = yes; 0 = *no*) in a given trial using a GLMM (binomial) with reproductive status, trial number in phase, and reproductive status × trial number in phase as predictor variables and fish identity as a random variable.
- Whether fish who make a choice are successful in their first disk push (1 = success; 0 = *fail*), using a GLMM (binomial) with reproductive status, trial number in phase, and reproductive status × trial number in phase as predictor variables and fish identity as a random variable.

All data visualization was done using the *ggplot2* package in Rstudio and Adobe Illustrator (Wickham 2016; Adobe Inc. 2023). All final code, associated analyses, and descriptions of changes made to figures in Illustrator are provided in Appendix S4. Results on associative learning training are provided in Appendix S5 and will not be further discussed in this paper.

## 3 Results

### 3.1 Pregnant Fish Decline in Spatial Learning Performance, Exhibiting More Non‐choice Trials

**FIGURE 2:**
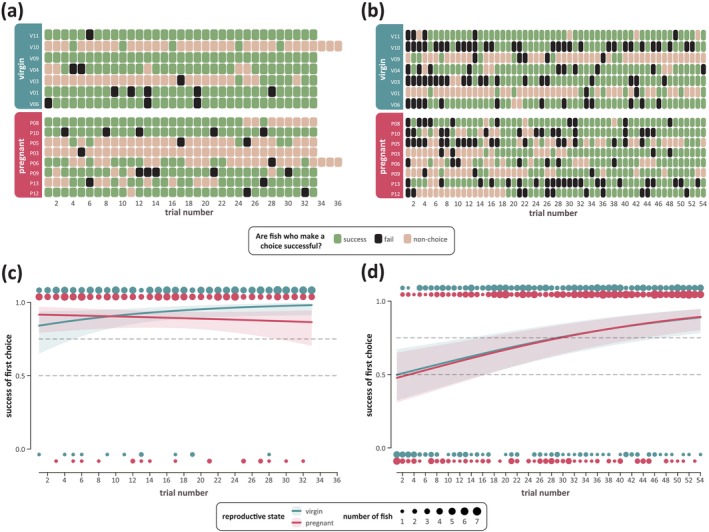
Behavioral results from the spatial and reversal learning tasks. (a,b) Heat‐maps representing the raw data from the spatial learning and reversal learning tasks, respectively, indicating the success of each fish (virgin or pregnant) on their first disk push. For both heat‐maps, all successes (1) are shown in green, failures (0) are shown in black, and nonchoice trials are shown in peach. (c,d) Spatial and reversal learning curves, respectively, as generated by our GLMMs predicting the success of the first disk push where all nonchoice trials are excluded. For both GLMM model predictions, lines indicate the model fit of the effect of the interaction variable (reproductive status (virgin) × trial number), with the lower and upper bounds of the IQRs indicated by the colored ribbons. Circles above and below the axes indicate the number of fish from each group who succeed (1) or fail (0) in each trial, where the area of the circle increases in proportion to the number of fish from 0 to 7. The horizontal dotted lines at 0.5 and 0.75 indicate when fish cross the 50% and 75% learning thresholds, respectively.

Both virgin and pregnant fish began the spatial learning task with ∼60% success and high variability between individuals. Fish from both groups also significantly increased the speed at which they make their first disk push over the course of the phase, regardless of whether this choice was correct or incorrect (Figure S5a and Table S2a). Raw data on success of the first disk push from both groups, as shown in Figure 2a, show that fish perform more non‐choice trials than true incorrect trials in this task, with virgin and pregnant fish having a 12:61 and 18:103 incorrect: non‐choice trial ratio, respectively.

To remove the impact that these non‐choice trials may have on our fish performance over time, we adjusted our model to only analyze trials in which fish made a choice, as shown in Figure 2c. Model predictions where non‐choice trials are included as failures or predicting whether or not fish make a choice are provided in Figure S4a,b and Table S1. With non‐choice trials removed, both virgin and pregnant fish exhibit high success over the course of the entire 33 trials, with both groups performing above the 75% learning threshold. However, there is a significant difference between performance over time between virgin and pregnant fish; virgin fish improved over the course of the phase, going from 84% ± 7% (SE) success at the start of the phase to 98% ± 2% (SE) at the end, while pregnant fish exhibited the opposite trend, declining from 91% ± 4% (SE) success to 86% ± 6% (SE; Table 1a).

**TABLE 1:** Outcomes from the statistical models for the (a) spatial and (b) reversal learning tasks.

	Estimate	SE	*z*‐value	*p*	
(a) Spatial learning: are fish who make a choice successful? (*n*_fish_ = 15, *n*_obs_ = 337) intercept	2.41	0.56	4.30	*p <* 0.001	***
Reproductive status (virgin)	−0.81	0.79	−1.03	0.30	
Trial number	−0.02	0.03	−0.62	0.54	
Reproductive status (virgin) × trial number	0.09	0.04	1.98	*p <* 0.05	*
(b) Reversal learning: are fish who make a choice successful? (*n*_fish_ = 15, *n*_obs_ = 608) intercept	−0.13	0.37	−0.36	0.72	
Reproductive status (virgin)	0.09	0.52	0.17	0.87	
Trial number	0.04	0.01	4.53	*p <* 0.001	***
Reproductive status (virgin) × trial number	−0.002	0.01	−0.18	0.86	

*Note: n*_fish_ = the number of fish analyzed; *n*_obs_ the number of observations. Significance is marked with stars where *p <* 0.001 is ***, *p <* 0.01 is **, and *p <* 0.05 is *.

Abbreviation: SE, standard error.

### 3.2 Virgin and Pregnant Fish Who Make A Choice Do Not Differ in Their Reversal Learning Performance

As expected, both virgin and pregnant fish start the reversal learning task with success rates below 50% (virgin: 32% ± 8% [SE]; pregnant: 22% ± 7% [SE]) as fish have to unlearn their previously enforced behavior in order to learn the new location of the food reward. Unlike the spatial learning task, fish did not significantly increase the speed of their first disk push (regardless of whether that push was correct or incorrect) over the course of the 54 trials (Figure S5b and Table S2b). As with the spatial learning task, raw data from both groups, as shown in Figure 2b, indicate that fish still have a high proportion of non‐choice trials, with virgin and pregnant fish having a 85:94 and 91:108 incorrect:non‐choice ratio, respectively.

To remove the impact of non‐choice trials on our assessment of fish performance, we adjusted our model to exclude all non‐choice trials, as shown in Figure 2d. Again, model predictions where non‐choice trials are included as failures or predicting whether or not fish make a choice are provided in Figure S4c,d and Table S1. After removing non‐choice trials, the model fit for both virgin and pregnant fish becomes almost the same: Both groups significantly increase their success performance over time, and the groups do not significantly differ from each other (Table 1b).

### 3.3 Neuronal Proliferation Zones in *P. gracilis*

**FIGURE 3:**
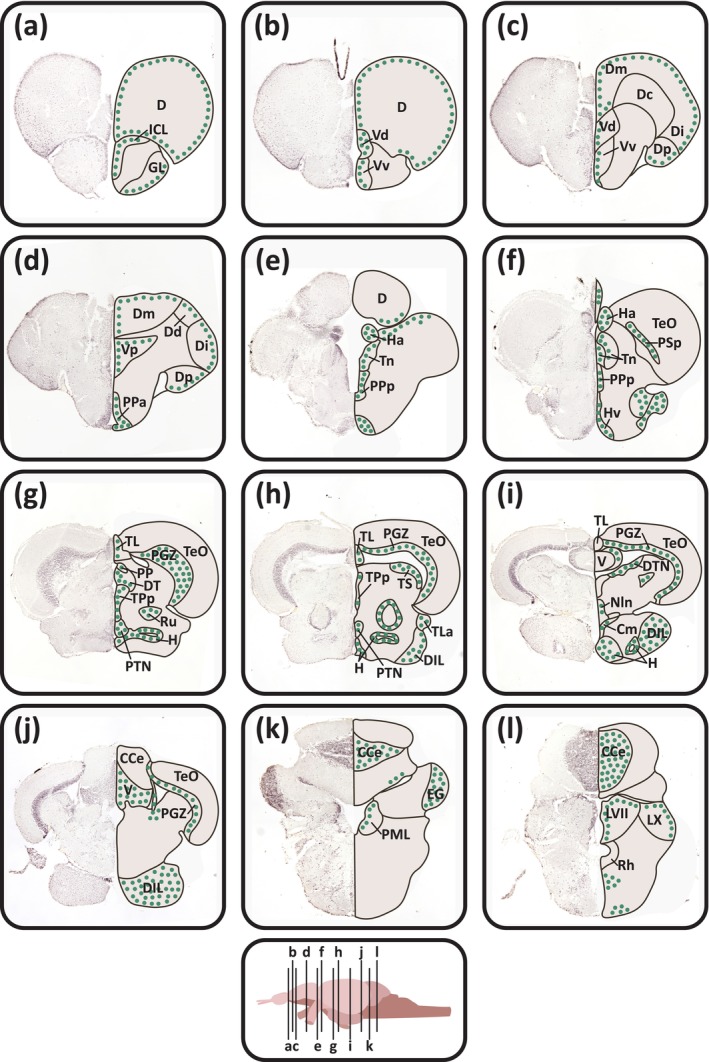
Neuronal proliferation zones in *Poeciliopsis* *gracilis* : (a–l) Left side of images: coronal sections of a virgin *P. gracilis* brain with *ki67+* cells (dark gray). Right side of images: schematic depiction of the identified brain regions (based on *Danio rerio* neuroanatomical atlas by Wulliman et al. (1996)), with green dots indicating the proliferative regions as identified with *ki67+* IHC staining. Legend below shows a schematic lateral view of the brain with lines to indicate approximate coronal positioning of the respective brain slice. High‐magnification images are provided in Appendix S5. Abbreviations: Cce (corpus cerebelli); Cm (corpus mamillare); D (dorsal telencephalon); Dc (dorsocentral telencephalon); Dd (dorsal region of dorsal telencephalon); Di (dorsolateral telencephalon); DIL (diffuse nucleus of the inferior lobe); Dm (dorsomedial telencephalon); Dp (dorsoposterior telencephalon); DT (dorsal thalamus); EG (eminentia granularis); GL (olfactory bulb, glomerular layer); H (hypothalamus); Ha (habenular nucleus); Hv (periventricular hypothalamus, ventral); ICL (olfactory bulb, internal cellular layer); LVII (lobus facialis); LX (lobus vagus); NLn (nucleus interpeduncularis); PGZ (periventricular gray zone of the TeO); PML (posterior mesencephalic lamina); PP (periventricular pretectal nucleus); PPa (parvocellular preoptic nucleus, anterior); PPp (parvocellular preoptic nucleus, posterior); PSp (parvocellular superficial pretectal nucleus); PTN (posterior tuberal nucleus); Rh (ventricular zone of rhombencephalic ventricle); RU (nucleus ruber); TeO (optic tectum); TL (torus longitudinalis); TLa (torus lateralis); Tn (thalamic nucleus); TPp (periventricular nucleus of posterior tuberculum); TS (torus semicircularis); V (valvula cerebelli); Vd (ventral telencephalon, dorsal nucleus); Vp (ventral telencephalon, postcomissural nucleus); Vv (ventral telencephalon, nucleus).

Before quantifying differences in the *ki67+* coverage between our virgin and pregnant females, we first had to visualize and map the baseline neuronal proliferation zones in virgin *P. gracilis* females which did not undergo behavioral testing. Figure 3 shows 12 consecutive coronal slices of the *P. gracilis* brain stained with the cell proliferation marker *ki67* and then schematically represented to highlight the proliferation zones.

### 3.4 Pregnant Females Have Decreased Neuronal Cell Proliferation in Some Brain Regions

**FIGURE 4:**
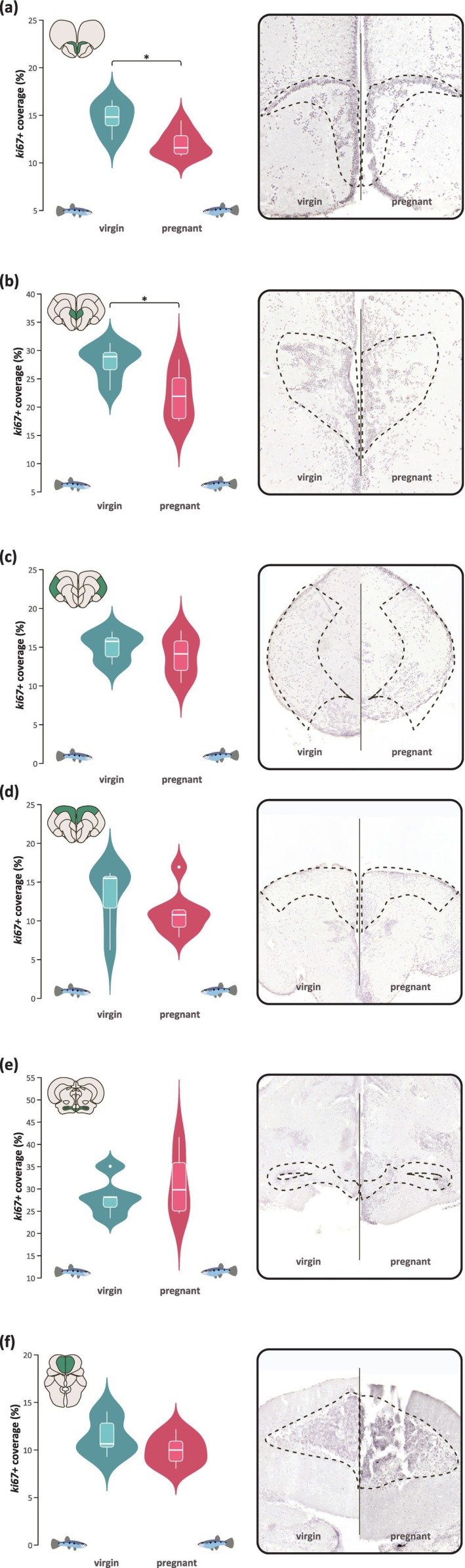
Insets show representative images from each group. (a) olfactory bulb, (b) ventral telencephalon, (c) dorsomedial telencephalon, (d) dorsolateral telencephalon, (e) hypothalamus, and (f) corpus cerebelli.

To determine if virgin and pregnant fish exhibited differential neuronal cell proliferation, we assessed percent *ki67+* coverage in a selection of the previously established *ki67+* brain regions (Figure 3): dorsomedial telencephalon, dorsolateral telencephalon, ventral telencephalon, olfactory bulb, hypothalamus, and corpus cerebelli. Figure 4 shows no significant difference between virgin and pregnant fish in percent *ki67+* coverage in the dorsomedial telencephalon (Figure 4a; *t* = 1.2137, df = 8.3256, *p*‐value = 0.2582), dorsolateral telencephalon (Figure 4b; *t* = 1.0955, df = 11.959, *p*‐value = 0.2949), hypothalamus (Figure 4e; *t* = −0.89809, df = 9.8858, *p*‐value = 0.3905), or corpus cerebelli (Figure 4f; *t* = 1.479, df = 9.4585, *p*‐value = 0.1716). However, there is a statistically significant decrease in percent *ki67+* coverage in pregnant fish compared to virgin fish in both the central telencephalon (Figure 4c; *t* = 2.8707, df = 10.54, *p*‐value = 0.01584) and the olfactory bulb (Figure 4d; *t* = 3.1506, df = 7.2481, *p*‐value = 0.01542).

## 4 Discussion

The aim of this study was to determine the impact of pregnancy in the lecithotrophic, viviparous fish, *P. gracilis* , on (1) maternal cognition and (2) cell proliferation in the maternal brain. Given lecithotrophic live‐bearers lack a placenta and do not provide nutrients to their offspring *during* pregnancy, their pregnancy is presumably less energetically demanding between fertilization and parturition compared to the pregnancy of placental species, like mammals. Our data show that pregnant *P. gracilis* do perform worse than virgin females in the spatial learning assay; however, this primarily stems from their reluctance to make any choices rather than an increased failure rate. Similarly, in the reversal learning task, pregnant females only differed from virgins in their hesitancy to make a choice rather than their success performance. In the brain, we found no differences in cell proliferation in the dorsal telencephalon, hypothalamus or corpus cerebelli. However, pregnant *P. gracilis* did exhibit a significant decrease in cell proliferation in the ventral telencephalon and olfactory bulb. Therefore, similar to mammals, pregnancy in the absence of a placenta in *P. gracilis* appears to modulate both fish behavior and neuronal cell proliferation.

### 4.1 Pregnant Fish Are Less Likely to Make a Choice

We sought to determine whether lecithotrophic live‐bearers like *P. gracilis* exhibit similar decreases in spatial memory performance and cognitive flexibility as those observed in mammals, despite experiencing a presumably less‐energetically costly pregnancy. However, true success frequency is difficult to compare between our two groups in both our behavioral tests because of the differential distribution of non‐choice trials. Therefore, while our models predict decreased success performance in pregnant fish in the spatial learning task and no impact on success performance in the reversal learning task, we consider the differential choice frequency between pregnant and virgin fish to be the main functional difference between the groups.

Decision‐making is a key part of executive functioning which includes cognitive flexibility, inhibitory control, and working memory. For fish, decision‐making is about making adaptive trade‐offs between different behaviors which impact fitness, such as predator avoidance, foraging, and reproduction/mating (Magnhagen and Magurran 2008). While research has shown in humans that pregnant women exhibit decreased executive functioning (Henry and Rendell 2007; Davies et al. 2018), which involves cognitive flexibility and may thereby impact decision‐making, our data indicate that while pregnant *P. gracilis* are still cognitively flexible they are still hesitant to make a choice. Given that our experiments were not specifically designed to measure choice‐propensity, future studies should repeat these studies with larger sample sizes and a wider variety of cognitive assays to further investigate the frequency of non‐choice trials and the overall cognitive performance of pregnant live‐bearing fish. Thus, the results of our study suggest an, albeit subtle, impact of pregnancy on maternal behavior and cognition in a live‐bearing species which lacks a placenta. While research in mammals has characterized a consistent and marked impact of pregnancy on maternal cognition (Davies et al. 2018; Henry and Rendell 2007; Barda et al. 2021; de Groot et al. 2006), our results indicate that this causal link may also be present in species like *P. gracilis* which have independently evolved live‐bearing outside of the mammalian evolutionary lineage. The comparatively subtle decrease in spatial learning and decision‐making we observe in *P. gracilis* may be due in part to the relatively low energetic demand *P. gracilis* experiences during pregnancy compared to placental live‐bearers like mammals. While pregnancy still poses a burden to *P. gracilis* females, changing their body shape/volume (Fleuren et al. 2018), increasing drag during swimming, and reducing their overall swimming performance (Quicazan‐Rubio et al. 2019), these fish are not additionally burdened by having to nourish their offspring during gestation like placental live‐bearers. Additionally, research has shown that superfetation—the ability to gestate multiple temporally overlapping broods—may have evolved in poeciliids like *P. gracilis* to offset some of the locomotor costs associated with pregnancy, further reducing the energetic burden of pregnancy for lecithotrophic, superfetatious species (Fleuren et al. 2019). In mammals, decreased spatial memory performance is especially present in late‐pregnancy when estradiol levels are high (Galea et al. 2000) and is often hypothesized to mark an energetic trade‐off in the maternal brain in preparation for post‐natal care behaviors (Ziomkiewicz et al. 2019; Sherry and Hampson 1997). While *P. gracilis* lacks maternal care and would therefore not require this energetic trade‐off, other lecithotrophic poeciliids have been shown to exhibit elevated estradiol during pregnancy compared to their placental counterparts (Barough et al. 2024) indicating that decreased spatial memory performance during pregnancy might be associated with estradiol levels, independent of maternal care. Taken together, our results indicate that pregnancy has a functionally minor impact on spatial memory performance in *P. gracilis* which may be related to the lower energetic burden of a superfetatious, lecithotrophic pregnancy, and their lack of maternal care. More research is needed to determine if estradiol levels may be driving this effect and whether this is conserved across other live‐bearing species with different reproductive strategies and varying amounts of postnatal maternal care.

### 4.2 Pregnant Fish Have Decreased Cell Proliferation in the Olfactory Bulb

Our study is the first to investigate cell proliferation in the brain of a poeciliid fish. Like other teleost fishes, *P. gracilis* shows widespread proliferation across the adult brain, including all the neurogenic regions in the “teleost ground plan of proliferation zones” which are generally conserved across species: the dorsal and ventral telencephalon (D & V respectively), olfactory bulb (ICL), hypothalamus (H), periventricular zone of the optic tectum (PGZ), and cerebellum (specifically the corpus cerebelli; CCe) (Ganz and Brand 2016; Zupanc 2011). Neuroanatomical research across other teleost species has provided evidence that these neurogenic regions represent both homologous neurogenic niches to those found in mammals (D & V), as well as new adult neurogenic niches unique to teleosts (H, PGZ, & CCe; Zupanc (2011)). In terms of homology, proliferating cells in the dorsal telencephalon (particularly the dorsolateral region; Di) represent one of the major neurogenic niches in mammals, sharing neurogenic markers and functionality with neuroprogenitors found in the mammalian hippocampus, causing researchers to widely consider these regions to be homologous (Ganz and Brand 2016; Zupanc 2011, 2021). Conversely, neurogenesis in the ventricular zone of the ventral telencephalon (Vv) represents the second mammalian neurogenic niche, with proliferating cells migrating tangentially from the Vv to the olfactory bulb, similar to the rostral migration of proliferating cells from the subventricular zone (SVZ) to the olfactory bulb in mammals (Ganz and Brand 2016; Adolf et al. 2006; Kishimoto et al. 2011). We chose to compare cell proliferation in our fish in the teleostean homologs of the mammalian proliferative zones, the ventral and dorsal telencephalon, as well as the olfactory bulb (as part of the ventral telencephalic migratory stream), the hypothalamus, and the corpus cerebelli.

We analyzed dorsal telencephalic cell proliferation by quantifying the medial and lateral portions separately, given that the latter region is most associated with the mammalian DG of the hippocampus (Ganz and Brand 2016). However, we found no significant difference between virgin and pregnant females in either dorsomedial or dorsolateral telencephalic cell proliferation (Figure 4a,b). While these results are statistically nonsignificant, there was a slight reduction in cell proliferation in pregnant females, particularly in the dorsomedial telencephalon, which may explain their decreased performance in the behavioral tasks. The low sample sizes in our study make it possible for a Type II statistical error, and larger sample sizes would be needed to determine if there is a statistically significant decrease in these regions. While we did not find statistically significant differences in the dorsal telencephalon, we did find a significant decrease in cell proliferation in both the ventral telencephalon and the olfactory bulb, indicating that pregnancy in *P. gracilis* may only modulate the proliferative phase of neurogenesis in the SVZ‐homologous neurogenic niche. Given that our study only assesses brain cell proliferation and is not able to confirm cell survival or differentiation, further lineage tracing studies are needed to confirm whether the altered cell proliferation we observe is indeed reflective of neurogenic capacity.

In rodents, pregnancy has been shown to increase neurogenesis in the SVZ, resulting in increased cell migration to the olfactory bulb, which promotes maternal odor discrimination and olfactory reception important for postpartum maternal care (Shingo et al. 2003; Medina and Workman 2020). Poeciliid fishes, like *P. gracilis* , notably lack postpartum maternal care and therefore do not require the same olfactory recognition of their offspring as mammals do. However, olfactory reception and discrimination is still important for pregnant fish given that many fish rely on olfactory reception to guide a variety of essential behaviors, including foraging/feeding, predator avoidance, defense, mate choice, and migration (Hara 1975; Kasumyan 2004). Our results indicate that female fish might experience altered olfactory bulb function during pregnancy, affecting olfactory reception and discrimination (Kermen et al. 2013). Given that pregnant females also exhibited a subtle decrease in choice propensity in our behavioral tasks, we tentatively propose a link between decreased ventral telencephalic and olfactory bulb cell proliferation and the performance of our pregnant fish in the spatial and reversal learning tasks. However, further research is needed to specifically test olfactory sensory performance of pregnant females to further investigate this potential relationship. Our results indicate that, similar to mammals, pregnancy decreases neuronal cell proliferation in *P. gracilis* , but unlike in mammals, we only find decreased cell proliferation in the SVZ‐homologous neurogenic niche. These results support a link between pregnancy and altered brain cell proliferation across different live‐bearing evolutionary lineages, which may be further influenced by reproductive strategy and maternal investment. Further research should take advantage of the reproductive diversity of live‐bearing lineages like the family Poeciliidae to better understand the link between pregnancy and neurogenesis.

## 5 Conclusions

Our study is the first to investigate the impact of pregnancy on maternal cognition and cell proliferation in a live‐bearing fish ( *Poeciliopsis gracilis* ) which lacks a placenta. Here, we also present the first overview of proliferation zones in a poeciliid fish. Our data indicate that pregnant females exhibit a subtle decrease in spatial learning performance and no functional difference in cognitive flexibility, instead exhibiting increased hesitancy to make a choice rather than a higher failure rate in both tasks. These findings correlate with our assessments of the brain, wherein we found no difference in cell proliferation in the dorsal telencephalon (the hippocampal analagous region). Instead, we found decreased cell proliferation in the ventral telencephalon and olfactory bulb, indicating that pregnant females may experience decreased olfactory reception which we propose may contribute to their choice‐aversion during the behavioral tasks. Future research in poeciliids is needed to further elucidate maternal olfactory reception and the role it might play in driving maternal decision‐making. Additionally, this study only investigates the cell proliferation phase of neurogenesis, and future studies should build upon this work to make a comprehensive overview of neurogenesis (incl. neuronal differentiation and maturation) in poeciliid fishes. Overall, our study indicates that pregnancy in *P. gracilis*, despite not having a placenta, does influence maternal behavior and neuronal cell proliferation.

## Author Contributions

**Tiffany R. Ernst:** conceptualization, methodology, data curation, formal analysis, visualization, investigation, writing – original draft, project administration, validation, writing – review and editing. **Alianne Keijzer:** methodology, investigation, data curation. **Sofia Vellere:** methodology, data curation, investigation. **Anthony Lee:** methodology, data curation, investigation. **Johan L. van Leeuwen:** conceptualization, methodology, supervision, resources, writing – review and editing. **Alexander Kotrschal:** conceptualization, methodology, supervision, resources, writing – review and editing. **Aniko Korosi:** conceptualization, writing – review and editing, project administration, resources, supervision, methodology. **Bart J. A. Pollux:** project administration, resources, supervision, writing – review and editing, funding acquisition, conceptualization, methodology.

## Funding

This study was funded by a Vidi grant (864.14.008) from the Nederlandse Organizatie voor Wetenschappelijk Onderzoek (NWO) awarded to B.J.A.P.

## Conflicts of Interest

The authors declare no conflicts of interest.

## Supporting information


**Figure S1:** Schematic of the tanks where fish were housed before and during behavioral trials. (a) A 3D depiction of the home tanks where fish were housed prior to behavioral testing. Each tank consisted of one compartment within a six‐compartment structure, allowing fish to see neighboring fish. These tanks were identical in size and shape to the behavioral tanks but lacked the necessary components for behavioral testing. (b) A top–down schematic of each home tank. (c) A 3D depiction of the experimental tanks where fish were housed during behavioral testing. These tanks contained a pair of guillotine doors which separated the home compartment from the testing arena. These doors could be open, partially open (allowing fish to look through the transparent door), or closed. (d) A top–down schematic of each experimental tank indicating the home compartment and the testing arena
**Figure S2:** Morphological characteristics of the fish in this study as measured at the end of the experiment, where *n* = 7 for virgin fish and *n* = 8 for pregnant. (a) Weight of the fish in grams (*t* = −0.26, df = 11.10, *p*‐value = 0.80). (b) Standard length of the fish in millimeters (*t* = −1.29, df = 10.99, *p*‐value = 0.22).
**Figure S3:** Training performance in habituation and associative learning phases. (a) An overview of the number of trials fish performed during each phase of the behavioral experiments, including the following: habituation, four well training, one well training, task testing, and reversal learning. Fish identities are listed along the *y*‐axis in descending order from lowest total number of trials to highest total number of trials per group (virgin or pregnant). Fish excluded from further analysis is marked with an X. (b) The number of trials fish needed to complete all training phases (habituation and training). Box plots indicate the median (virgin: 132 trials, pregnant: 170 trials) and the interquartile range (IQR; virgin: 51, pregnant: 24). Underlain violin plots represent the distribution of the fish, where *n* = 7 for virgin and *n* = 8 for pregnant.
**Figure S4:** Preliminary behavioral GLMMs generated during model testing for the spatial and reversal learning tasks. (a, c) Spatial and reversal learning curves, respectively, as generated by our GLMMs, predicting the success of the first disk push where non‐choice trials are treated as failures. (b, d) Spatial and reversal learning curves, respectively, as generated by our GLMMs, predicting whether or not fish make a choice in each trial where any disk push is a success and non‐choice is a fail. For all GLMM model predictions, lines indicate the model fit of the effect of the interaction variable (reproductive status (virgin) × trial number), with the lower and upper bounds of the IQRs indicated by the colored ribbons. Circles above and below the axes indicate the number of fish from each group who succeed (1) or fail (0) in each trial, where the area of the circle increases in proportion to the number of fish from 0 to 7. The horizontal dotted lines at 0.5 and 0.75 indicate when fish cross the 50% and 75% learning thresholds, respectively.
**Figure S5:** Heat‐maps representing the time until the first disk push (regardless of success) per fish for the spatial and reversal learning tasks. (a) The time until the first disk push for each fish (virgin or pregnant) in the spatial learning task where time is represented by a gradient scale from teal to chartreuse with times < 2 min in shades of teal and times > 2 min in shades of chartreuse. All non‐choice trials are shown in peach. (b) The time until the first disk push for each fish (virgin or pregnant) in the reversal learning task where time is represented by a gradient scale from teal to chartreuse with times < 2 min in shades of teal and times > 2 min in shades of chartreuse. All non‐choice trials are shown in peach.
**Table S1:** Outcomes from the statistical model testing for the (a, b) spatial and (c, d) reversal learning tasks. *n*_fish_ = the number of fish analyzed; *n*_obs_ the number of observations. SE = standard error. Significance is marked with stars where: *p* < 0.001 is ***, *p* < 0.05 is *, and *p* < 0.1 is ・.
**Table S2:** Outcomes from the statistical models for time (min) until the first push for both the spatial and reversal learning tasks.*n*_fish_ = the number of fish analyzed; *n*_obs_ the number of observations. SE = standard error. Significance is marked with stars where: *p* < 0.001 is ***, *p* < 0.05 is *, and *p* < 0.1 is ・.

**Appendix S1:** Liver paste preparation protocol.
**Appendix S2:** Extended methods.

## Data Availability

The data, supplemental methods, code, and analyses that support the findings of this study are openly available in Figshare at 10.6084/m9.figshare.26042638 (data) and 10.6084/m9.figshare.26042641 (supplemental methods and code and analyses).
