# Supplementary material for: The Impacts of Pregnancy on Cognition and Cell Proliferation in a Live‐Bearing Fish ( Poeciliopsis gracilis )
Source: Eur J Neurosci. 2026 May 8;63:e70523. doi: 10.1111/ejn.70523 (PMC13155941; doi:10.1111/ejn.70523)
Supplement: Supplementary file 1 — Figure S1: Schematic of the tanks where fish were housed before and during behavioral trials. (a) A 3D depiction of the home tanks where fish were housed prior to behavioral testing. Each tank consisted of one compartment within a six‐compartment structure, allowing fish to see neighboring fish. These tanks were identical in size and shape to the behavioral tanks but lacked the necessary components for behavioral testing. (b) A top–down schematic of each home tank. (c) A 3D depiction of the experimental tanks where fish were housed during behavioral testing. These tanks contained a pair of guillotine doors which separated the home compartment from the testing arena. These doors could be open, partially open (allowing fish to look through the transparent door), or closed. (d) A top–down schematic of each experimental tank indicating the home compartment and the testing arena Figure S2: Morphological characteristics of the fish in this study as measured at the end of the experiment, where n = 7 for virgin fish and n = 8 for pregnant. (a) Weight of the fish in grams (t = −0.26, df = 11.10, p‐value = 0.80). (b) Standard length of the fish in millimeters (t = −1.29, df = 10.99, p‐value = 0.22). Figure S3: Training performance in habituation and associative learning phases. (a) An overview of the number of trials fish performed during each phase of the behavioral experiments, including the following: habituation, four well training, one well training, task testing, and reversal learning. Fish identities are listed along the y‐axis in descending order from lowest total number of trials to highest total number of trials per group (virgin or pregnant). Fish excluded from further analysis is marked with an X. (b) The number of trials fish needed to complete all training phases (habituation and training). Box plots indicate the median (virgin: 132 trials, pregnant: 170 trials) and the interquartile range (IQR; virgin: 51, pregnant: 24). Underlain violin plots represent the dis [file EJN-63-0-s002.pdf]

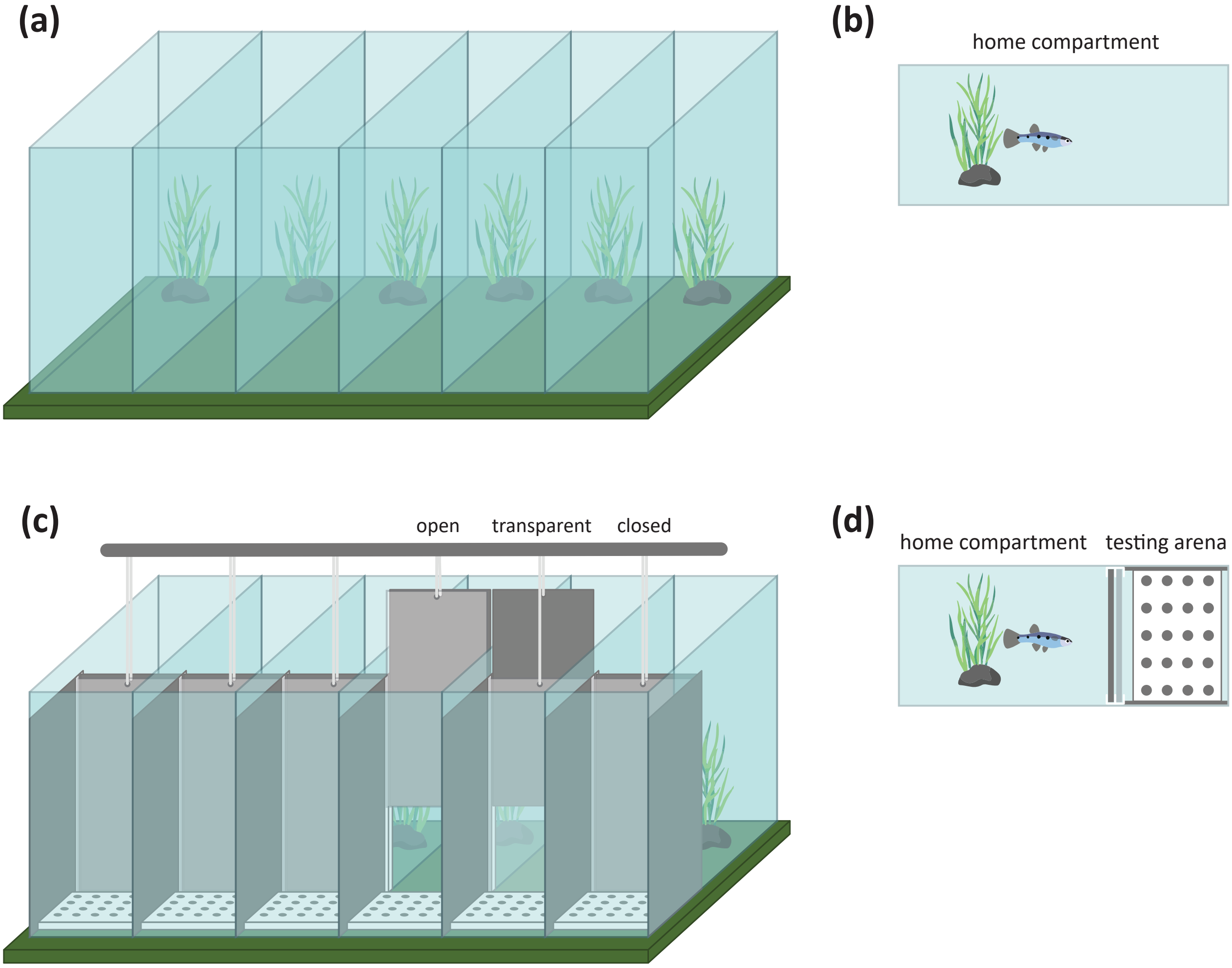

Figure S1. Schematic of the tanks where fish were housed before and during behavioral trials. (a) A 3D depiction of the home tanks where fish were housed prior to behavioral testing. Each tank consisted of one compartment within a 6 compartment structure, allowing fish to see neighboring fish. These tanks were identical in size and shape to the behavioral tanks but lacked the necessary components for behavioral testing. (b) A top-down schematic of each home tank. (c) A 3D depiction of the experimental tanks where fish were housed during behavioral testing. These tanks contained a pair of guillotine doors which separated the home compartment from the testing arena. These doors could be open, partially open (allowing fish to look through the transparent door), or closed. (d) A top-down schematic of each experimental tank indicating the home compartment and the testing arena.

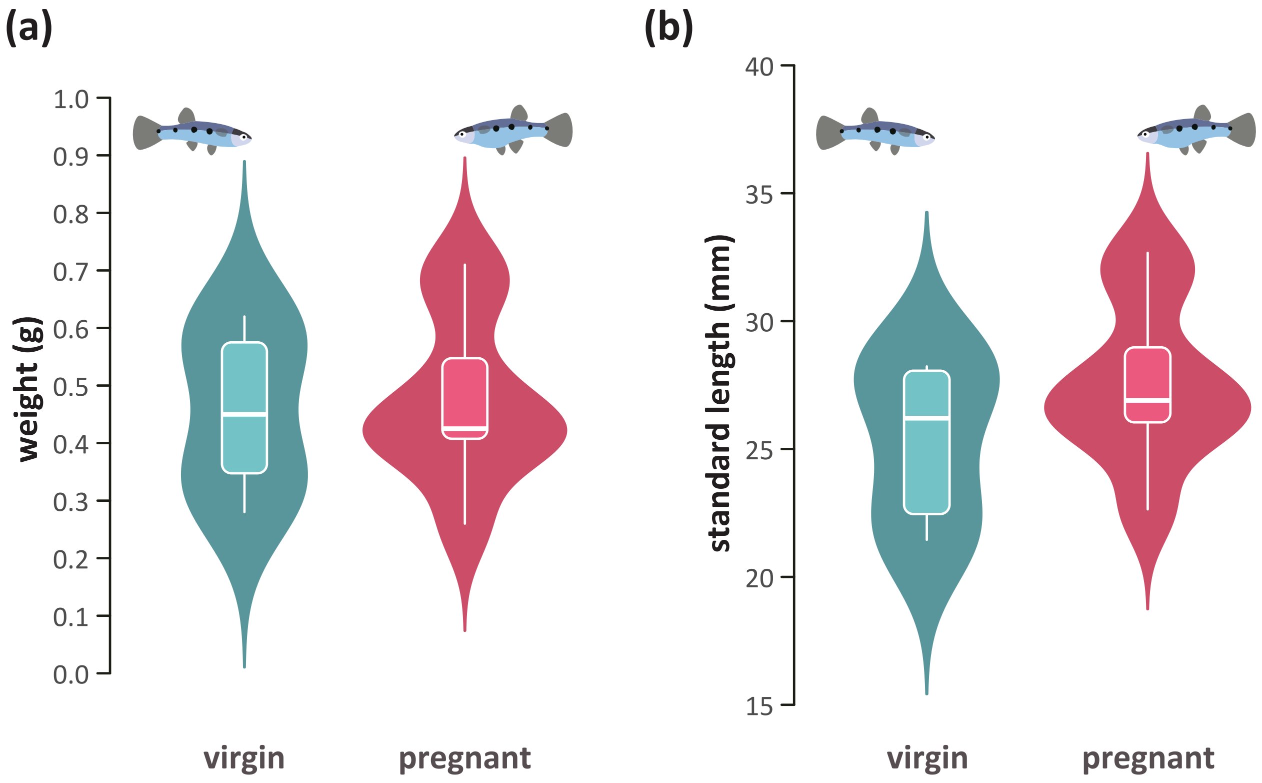

Figure S2. Morphological characteristics of the fish in this study as measured at the end of the experiment, where n = 7 for virgin fish and n = 8 for pregnant. (a) weight of the fish in grams ($t = -0.26$, $df = 11.10$, p-value = 0.80). (b) standard length of the fish in millimeters ($t = -1.29$, $df = 10.99$, p-value = 0.22).

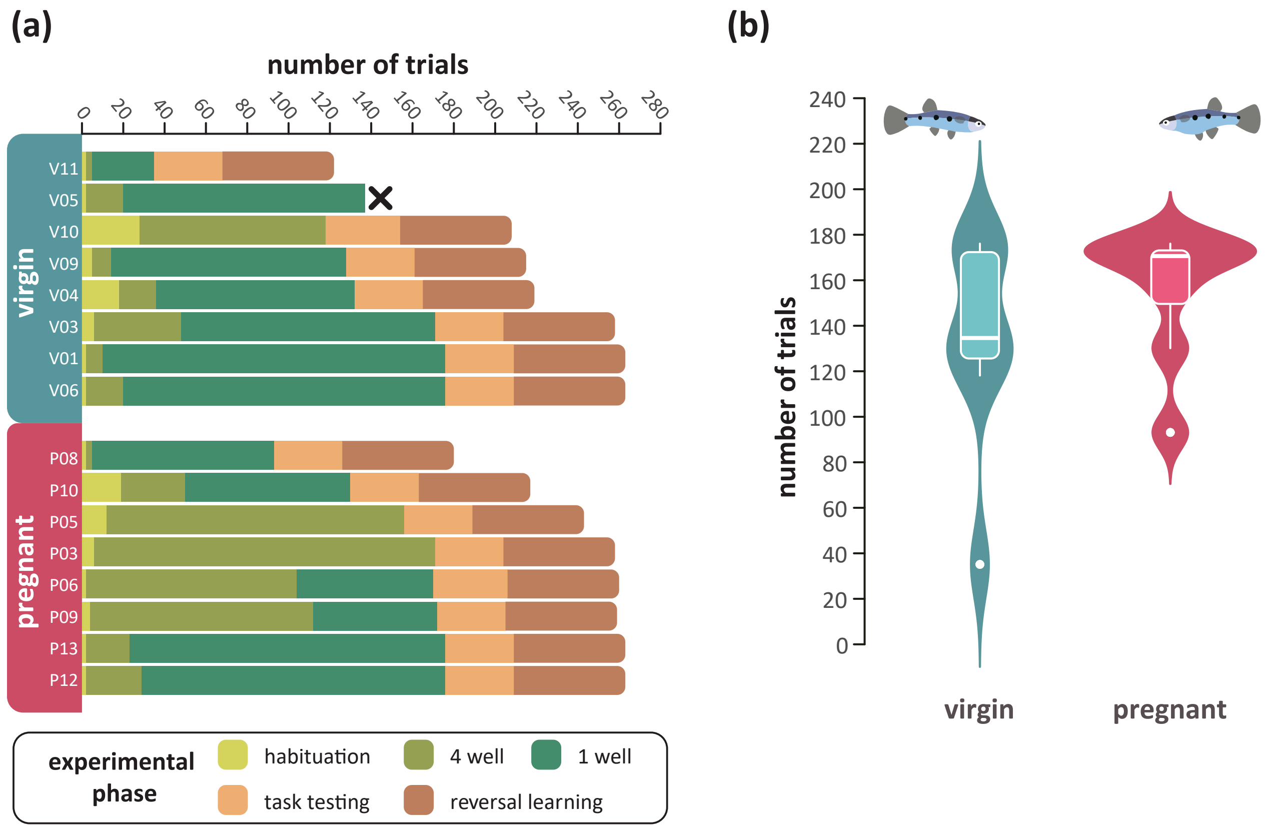

Figure S3. Training performance in habituation & associative learning phases. (a) An overview of the number of trials fish performed during each phase of the behavioral experiments, including: habituation, 4 well training, 1 well training, task testing, and reversal learning. Fish identities are listed along the y-axis in descending order from lowest total number of trials to highest total number of trials per group (virgin or pregnant). Fish excluded from further analysis is marked with an X. (b) The number of trials fish needed to complete all training phases (habituation & training). Box plots indicate the median (virgin: 132 trials, pregnant: 170 trials) and the inter-quartile range (IQR; virgin: 51, pregnant: 24). Underlain violin plots represent the distribution of the fish, where n = 7 for virgin and n = 8 for pregnant.

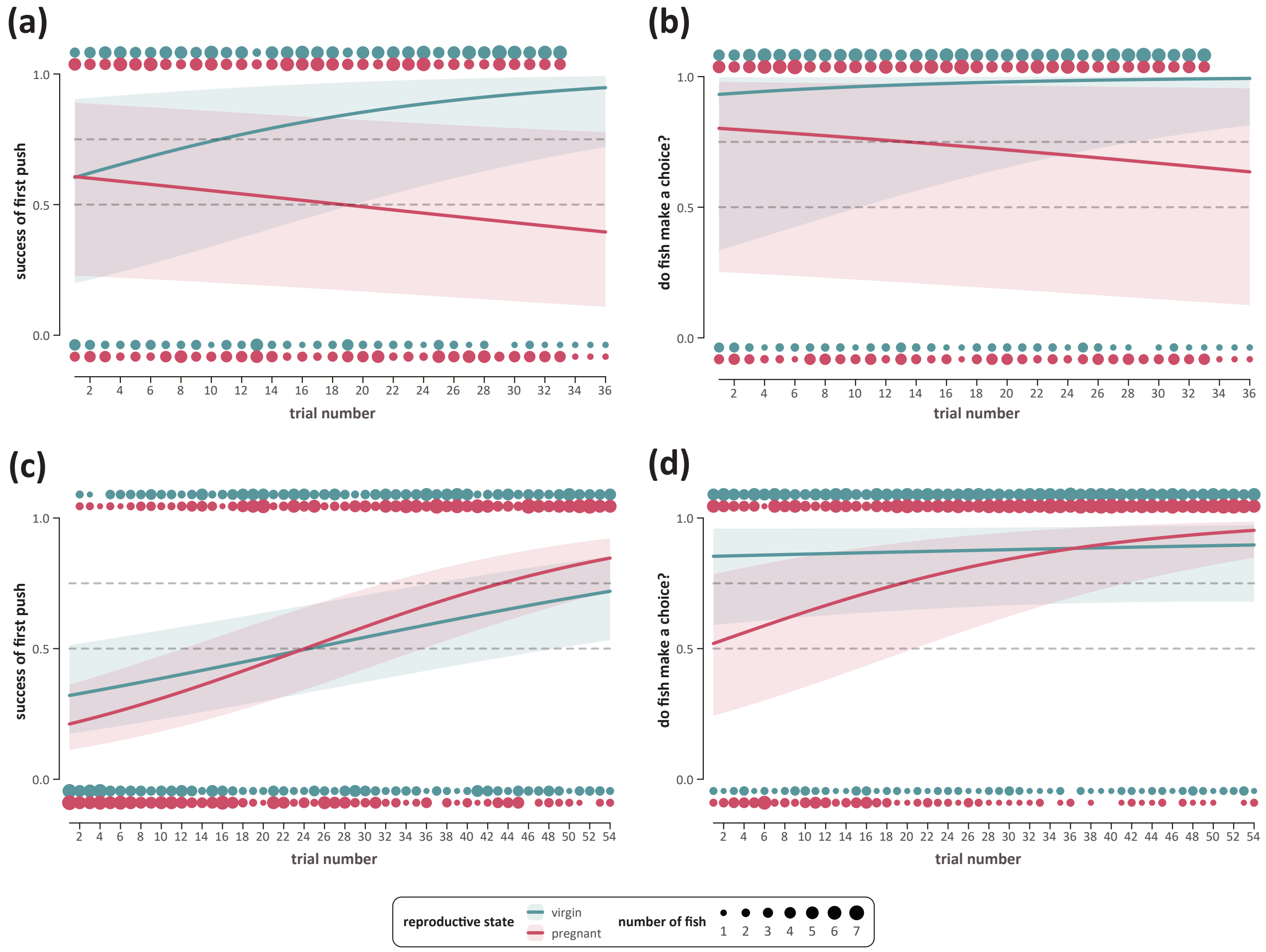

Figure S4. Preliminary behavioral GLMMs generated during model testing for the spatial and reversal learning tasks. (a) and (c) Spatial and reversal learning curves, respectively, as generated by our GLMMs, predicting the success of the first disk push where non-choice trials are treated as failures. (b) and (d) Spatial and reversal learning curves, respectively, as generated by our GLMMs, predicting whether or not fish make a choice in each trial where any disk push is a success and non-choice is a fail. For all GLMM model predictions, lines indicate the model fit of the effect of the interaction variable (reproductive status (virgin) × trial number), with the lower and upper bounds of the IQRs indicated by the colored ribbons. Circles above and below the axes indicate the number of fish from each group who succeed (1) or fail (0) in each trial, where the area of the circle increases in proportion to the number of fish from 0 to 7. The horizontal dotted lines at 0.5 and 0.75 indicate when fish cross the 50% and 75% learning thresholds, respectively.

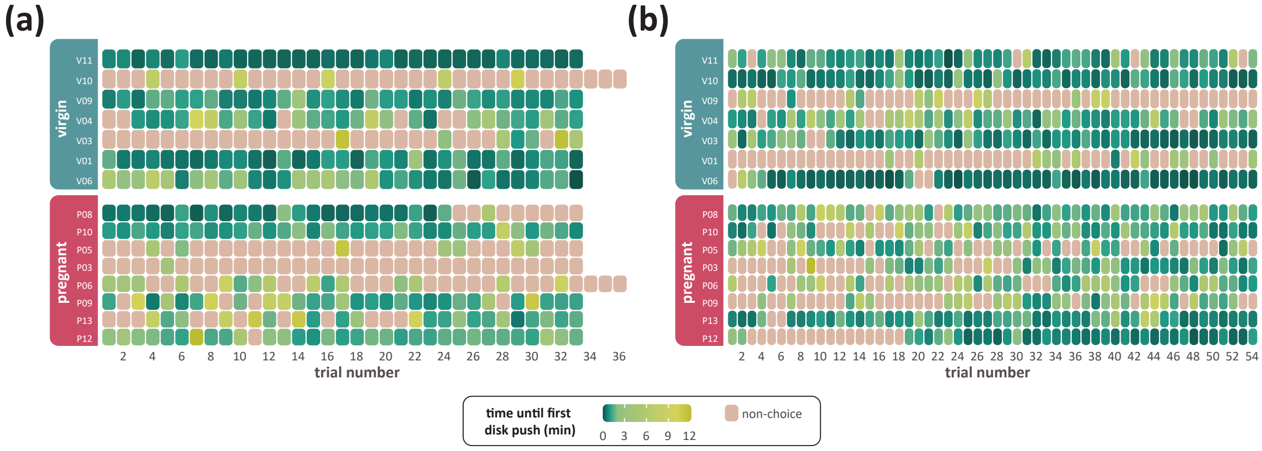

Figure S5. Heat-maps representing the time until the first disk push (regardless of success) per fish for the spatial and reversal learning tasks. (a) The time until the first disk push for each fish (virgin or pregnant) in the spatial learning task where time is represented by a gradient scale from teal to chartreuse with times $<$ 2 min in shades of teal and times $>$ 2 min in shades of chartreuse. All non-choice trials are shown in peach. (b) The time until the first disk push for each fish (virgin or pregnant) in the reversal learning task where time is represented by a gradient scale from teal to chartreuse with times $<$ 2 min in shades of teal and times $>$ 2 min in shades of chartreuse. All non-choice trials are shown in peach.

SUPPLEMENTARY TABLES

Table S1. Outcomes from the statistical model testing for the (a) & (b) spatial and (c) & (d) reversal learning tasks. n_{fish} = the number of fish analyzed; n_{obs} the number of observations. SE = standard error. Significance is marked with stars where: $p<0.001$ is ***, $p<0.05$ is *, and $p<0.1$ is ·.

	estimate	SE	z-value	p-value	
**Spatial learning:**					
**(a) success of the first disk push**					
(n_{fish}= 15, n_{obs} = 501)					
intercept	0.46	0.85	0.54	0.59	
reproductive status (virgin)	-0.12	1.26	-0.09	0.93	
trial number	-0.02	0.02	-1.53	0.13	
reproductive status (virgin) × trial number	0.10	0.03	3.43	p<0.001	***
**(b) do fish make a choice?**					
(n_{fish}= 15, n_{obs} = 501)					
intercept	1.42	1.27	1.12	0.26	
reproductive status (virgin)	1.13	2.08	0.54	0.59	
trial number	-0.02	0.02	-1.34	0.18	
reproductive status (virgin) × trial number	0.09	0.03	2.69	p<0.01	**
**Reversal learning:**					
**(a) success of the first disk push**					
(n_{fish}= 15, n_{obs} = 810)					
intercept	-1.37	0.39	-3.56	p<0.001	***
reproductive status (virgin)	0.59	0.57	1.04	0.30	
trial number	0.06	0.01	7.62	p<0.001	***
reproductive status (virgin) × trial number	-0.03	0.01	-2.32	p<0.05	*
**(b) do fish make a choice?**					
(n_{fish}= 15, n_{obs} = 810)					
intercept	0.02	0.62	0.04	0.97	
reproductive status (virgin)	1.73	0.95	1.82	0.07	.
trial number	0.06	0.01	6.28	p<0.001	***
reproductive status (virgin) × trial number	-0.05	0.01	-3.45	p<0.001	***

Table S2. Outcomes from the statistical models for time (min) until the first push for both the spatial and reversal learning tasks. n_{fish} = the number of fish analyzed; n_{obs} the number of observations. SE = standard error. Significance is marked with stars where: $p<0.001$ is ***, $p<0.05$ is *, and $p<0.1$ is ·.

	estimate	SE	z-value	p-value	
**(a) Spatial learning: time until first push**					
(n_{fish}= 15, n_{obs} = 335)					
intercept	0.75	0.22	3.34	p<0.001	***
reproductive status (pregnant)	-0.28	0.30	-0.94	0.35	
trial number	0.006	0.003	2.20	p<0.05	*
reproductive status (pregnant) × trial number	-0.003	0.003	-0.92	0.36	
**(b) Reversal learning: time until first push**					
intercept	0.56	0.13	4.28	p<0.001	***
reproductive status (pregnant)	-0.23	0.18	-1.31	0.19	
trial number	0.002	0.001	1.91	0.06	·
reproductive status (pregnant) × trial number	0.001	0.002	0.74	0.46	
