# Supplementary material for: The Impacts of Pregnancy on Cognition and Cell Proliferation in a Live‐Bearing Fish ( Poeciliopsis gracilis )
Source: Eur J Neurosci. 2026 May 8;63:e70523. doi: 10.1111/ejn.70523 (PMC13155941; doi:10.1111/ejn.70523)
Supplement: Supplementary file 2 — Appendix S1: Liver paste preparation protocol. Appendix S2: Extended methods. [file EJN-63-0-s001.pdf]

Appendix S1 – Liver Paste Preparation Protocol: The impacts of pregnancy on cognition and cell proliferation in a live-bearing fish (*Poeciliopsis gracilis*)

T.R. Ernst*, A. Keijzer, S. Vellere, A. Lee, A. Korosi, J.L. van Leeuwen, A. Kotrschal, & B.J.A. Pollux

* This protocol has been adapted from previous protocols by A. Hagmayer, M. Fleuren, & D. Reznick.

Ingredients:

- 1 kg Beef liver, deveined[1] (fresh or frozen)
- 900 ml water
- 473 ml baby food[2]
- 20 g table salt (NaCl)

Note: Amounts provided are for 1 kg of beef liver, however the recipe can be scaled according to the amount of liver you purchase.

Materials & Equipment

- metal sieve
- electric blender
- measuring cup
- kitchen scale
- 1 large mixing bowl
- 1 large mixing spoon
- 1 double boiler or two steel pots which can be used as a double boiler
- 3–4 soft silicon ice cube trays

Protocol:

1. If using frozen liver, defrost frozen liver in plastic bags in a room temperature water bath until fully defrosted.
2. Devein all liver pieces before cutting into small cubes (~ 2 cm^2)
3. Place the pitcher part of the blender on the kitchen scale and tare or zero the scale.
4. Add the liver pieces to the blender until it is max. 1/3 full. Take the weight of the liver in the blender and use it to calculate the amount of water/baby food/salt needed.
5. Slowly add the water keeping in mind that you cannot remove water later. Pulse the blender a few times before blending at a low speed for about 1 minute and then switch to a higher speed until the mixture is fully blended.
6. Place the sieve over the mixing bowl and pour the liver/water mixture in. You will need to "massage" the liver paste over the sieve with the back of the spoon to get the liquid to pass through and get past any of the small chunks.
7. Repeat steps 3–6 until all the liver is processed. Meanwhile, fill the bottom of the double boiler with water and bring to a boil.
8. Add 20 grams of salt per kg of liver to the liver/water mixture.
9. Add the baby food to the mixture and stir until fully combined.

[1]Beef liver can be purchased on request at most local butcher shops (**DO NOT** substitute with pork liver as this is not equivalent). If possible, calf's liver is preferred as this will have a lower chance of containing potential toxins; biological or organic beef liver is also recommended. Beef liver contains many blood vessels surrounded by strong white connective tissue. Therefore the all the liver pieces must be manually deveined with the connective tissue removed prior to making the liver paste to make it easier to sieve the final product.

[2]The baby food used should be a creamy paste without chunks. We recommend using Olvarit ™apple, pear and apricot blend for children ages 6+. Avoid using any baby food with added sugars or preservatives.

10. Add the liver mixture to the top of the double boiler until $\sim 2/3$ full. Place the top of the double boiler above the boiling water; make sure the water level in the lower put comes up to roughly the same height as the liver paste in the upper pot.
11. Reduce the heat on the water to a simmer and allow the liver mixture to simmer for about 30 minutes, or until all of the liver paste changes colour (should become more brown and less red). During this time the liver will expand and the water will separate out. You will need to stir the liver periodically to cook it but in the last stages leave the water separated so it is easy to pour off and remove.
12. Once the liver is cooked, pour off the separated water and run the final mixture through the sieve again to remove any chunks. Blend the cooked mixture one more to further smooth out the mixture. The consistency should be smooth and creamy (like tooth paste). If the mixture has become too dry you can always add a bit more water back again.
13. Repeat steps 10–12 until all the liver mixture is processed into a paste.
14. Scoop the final liver paste into the silicon ice cube trays. Cover the filled ice cube trays with plastic foil and leave to cool at room temperature.
15. Once cooled, put the ice cube trays in the freezer (-20°C) for storage until ready to use.

When ready to use, pop out the needed number of liver paste cubes and defrost in a clean beaker at room temperature. The desired amount of paste can then be administered to each tank using a syringe.

APPENDIX S2 – EXTENDED METHODS: THE IMPACTS OF PREGNANCY ON COGNITION AND CELL PROLIFERATION IN A LIVE-BEARING FISH (*POECILIOPSIS GRACILIS*)

T.R. Ernst, A. Keijzer, S. Vellere, A. Lee, A. Korosi, J.L. van Leeuwen, A. Kotrschal, & B.J.A. Pollux

Behavioral Training & Testing

Behavioral experiments were performed twice a day on weekdays, at 8:00 and 16:00 (to replace normal feeding times) and fish were no longer fed additional food at 12:00. During the behavioral trials, fish could consume a maximum of 90 μL of liver paste per testing period which was based on the $\sim$60 μL of liver paste fish consumed per day prior to the experiments.

Habituation

To reduce stress and enhance learning, fish were given a period of habituation as part of their training to acclimatize them to the wells-plate and the moving doors. After being placed in their experimental tanks, fish were given two days to become accustomed to the wells-plate while the doors were in place but left in the open position. During these two days fish were fed normally by the caretakers. After this period of acclimatization the doors were lowered and only opened during the training periods. The fish were then trained to eat from the wells-plate; at the start of each training period a 100 μL Hamilton pipette was used to administer 20 μL of liver paste to 4 adjacent wells and onto the plate in the middle of the four wells, as indicated in Figure 1. To begin the training the opaque door was lifted to let the fish inspect the testing arena through the transparent door for 10 seconds. Then the transparent door was lifted to allow the fish to enter the testing arena and eat the liver paste. The doors were left open for 2 hours after which they were closed and the wells-plate was emptied of any remaining liver paste. If the fish successfully ate the liver paste from the wells-plate within the 2 hr training period the liver paste was only administered inside the wells and not on top of the plate in the next training period (Figure 1). Once the fish had successfully eaten the liver paste from the wells within 30 min for two consecutive training periods the fish could continue into the training phases.

Training

During the training phases, the fish had to learn to associate the location of a green disk with the location of the liver paste. Each training period consisted of three consecutive trials which each lasted a maximum of 12 min. At the start of each training period, 30 μL ($\pm$ 3 μL) liver paste was divided evenly between four adjacent wells, as indicated in Figure 1. A green plastic disk was then positioned in the middle of these four wells (4 well sub-phase; Figure 1). To start each trial the opaque door was first opened for 10 seconds to allow the fish to inspect the testing arena. Then the transparent door was opened to allow the fish to enter the training arena to eat the liver paste. If the fish did not eat within 2 min, the doors were left open for a maximum of 10 additional min or until the fish had eaten from the wells-plate. If the fish successfully ate within 2 min for three consecutive trials, in the next trial the liver paste was only administered to one well, as indicated in Figure 1 (1 well sub-phase). For each consecutive trial, the green disk was moved closer to the well containing the liver paste until it completely covered the well. If the fish did not solve the task during the maximum 12 min, the disk was moved $\sim$5 mm to slightly uncover the liver paste for the next trial. Fish were trained in these phases until they were able to successfully dislodge the disk when it was fully covering the liver paste in at least 6 consecutive trials or until they had reached $\sim$30 days of behavioral training (habituation & training combined).

Spatial Learning

In the spatial learning task, fish had to localize the liver paste when presented with two identical green disks in different locations. Both disks concealed a food reward to prevent the fish from relying

on olfactory cues to find the liver paste (Hara, 1975). As shown in Figure 1, the left disk remained the rewarded disk where the disk could be moved to uncover the food reward while the right disk was fixed in place with a foam plug. Each trial began by opening the opaque door for 10 seconds to allow the fish to observe the training arena and then the transparent door was opened to allow the fish to enter the training arena. Fish were given 2 min to solve the task and were scored based on the success of their first disk push. A first push on the rewarded desk was scored as correct (1) and a first push on the unrewarded disk was scored as incorrect (0). If fish did not push either disk within 2 min, the trial was scored as non-choice (NC). For incorrect and non-choice trials, the fish were left to solve the task for an additional 10 min, as described in the training. Fish were considered to have learned the task once they had $> 75\%$ correct choices over at least six consecutive trials. Fish performed a maximum of 33 trials in the spatial learning task (with the exception of one experimental block – V10 and P3 – which received 3 extra trials due to a counting error) before moving on to the task reversal. The number of trials (33) was determined based on the number of trials it took for the best performing fish (V11) to pass the 75% learning threshold.

Task Reversal

The task reversal phase of the behavioral testing was the same as the spatial learning task except that the location of the rewarded and unrewarded disks were swapped, as shown in Figure 1. These trials were conducted and scored the same way as the spatial learning but fish performed 54 trials of task reversal before ending the experiment; again, this number of trials was determined based on the number of trials it took for the most successful fish (V11) to reach $> 75\%$ correct choices over at least six consecutive trials.

APPENDIX S5 – EXTENDED RESULTS: THE IMPACTS OF PREGNANCY ON COGNITION AND CELL PROLIFERATION IN A LIVE-BEARING FISH (*POECILIOPSIS GRACILIS*)

T.R. Ernst, A. Keijzer, S. Vellere, A. Lee, A. Korosi, J.L. van Leeuwen, A. Kotrschal, & B.J.A. Pollux

Associative learning is similar for virgin and pregnant fish

In the training phases, the majority of fish (5/6 virgin & 7/8 pregnant) were transitioned directly from the training period into the spatial learning task after ∼30 days of training because they were unable to pass the training criteria on their own; only fish P08 and V11 were able to complete 6 consecutive 1 well trials to pass on their own from the training to the task testing. Supplementary Figure S3a shows the number of trials performed by each fish in each of the different phases of the behavioral testing, where there is no substantial difference between the groups. Additionally, there was no significant difference between virgin and pregnant fish in the number of trials fish needed to complete all of the training phases (Wilcoxon ranked sum test; $n = 7$ virgin & $n = 8$ pregnant, $W = 22.5$, p-value $= 0.5589$; Supplementary Figure S3b).

ki67+ Cell Proliferation Atlas for *Poeciliopsis gracilis*

presented as part of

APPENDIX S5 - EXTENDED RESULTS: THE IMPACTS OF PREGNANCY ON COGNITION AND CELL PROLIFERATION IN A LIVE-BEARING FISH (*POECILIOPSIS GRACILIS*)

T.R. Ernst, A. Keijzer, S. Vellere, A. Lee, A. Korosi, J.L. van Leeuwen, A. Kotrschal, & B.J.A. Pollux

Brain regions were defined in this atlas by comparing morphological characteristics of our brain slices after either Nissl or *ki67*+ staining to established and annotated brain atlases for *Danio rerio* (Wulliman et al., 1996) and *Poecilia reticulata* (Fischer et al., 2018).

Caption for all images in this atlas:
Figure 3. Neuronal proliferation zones in P. gracilis: (a–l) Left side of images: coronal sections of a virgin *P. gracilis* brain with *ki67*+ cells (dark grey). Right side of images: schematic depiction of the identified brain regions (based on *D. rerio* neuroanatomical atlas by Wulliman et al. (1996)), with green dots indicating the proliferative regions as identified with *ki67*+ IHC staining. Legend below shows a schematic lateral view of the brain with lines to indicate approximate coronal positioning of the respective brain slice.

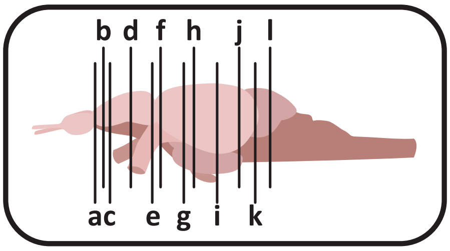

Fischer, E. K., Westrick, S. E., Hartsough, L., and Hoke, K. L. (2018). Differences in neural activity, but not behavior, across social contexts in guppies, poecilia reticulata. Behavioral ecology and sociobiology, 72:1–12.

Wulliman, M. F., Rupp, B., and Reichert, H. (1996). Neuroanatomy of the zebrafish brain: a topological atlas. Birkhäuser.

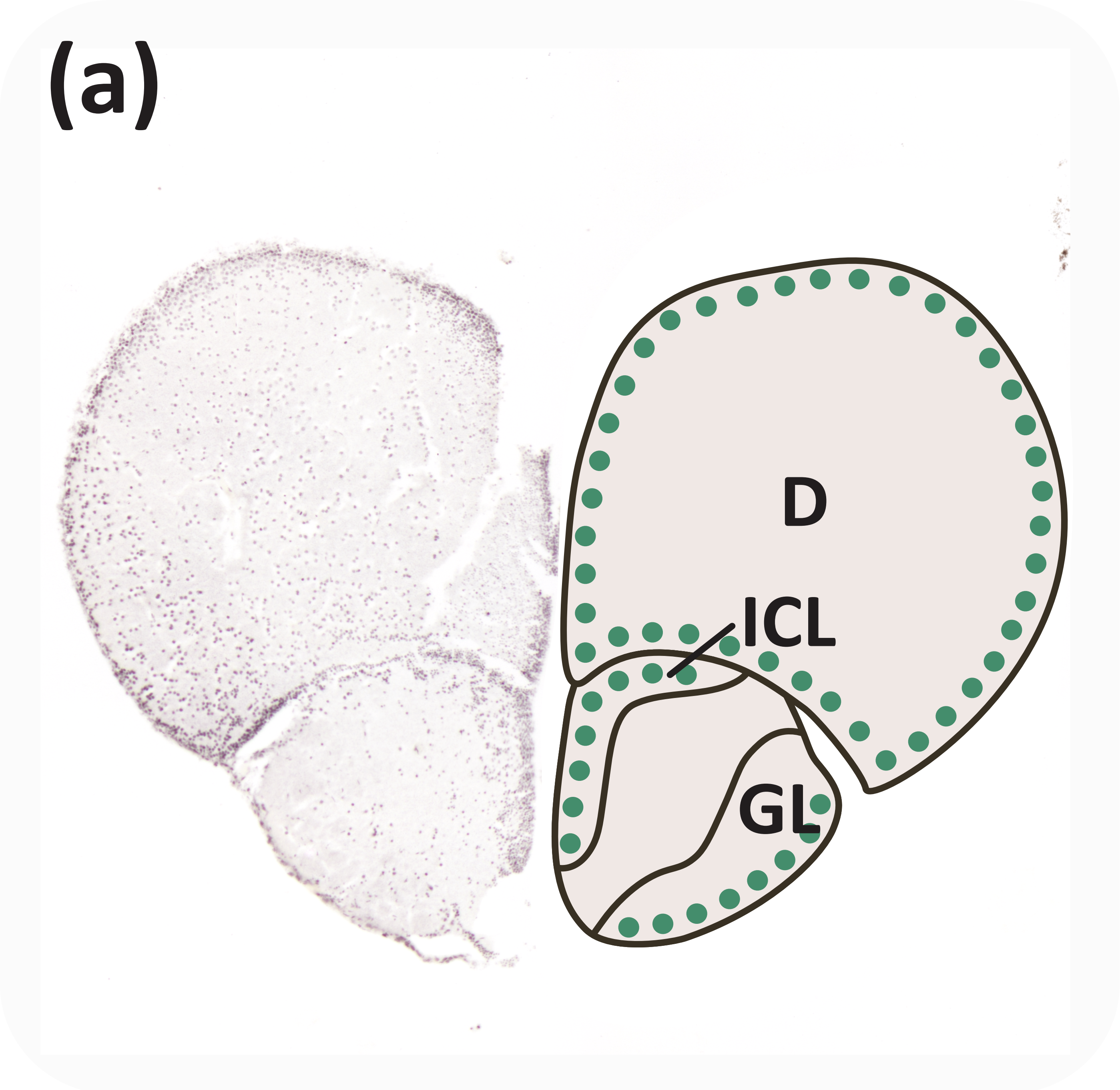

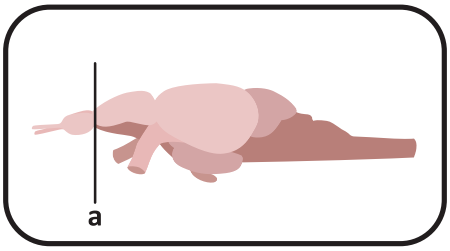

Abbreviations: D (dorsal telencephalon); GL (olfactory bulb, glomerular layer); ICL (olfactory bulb, internal cellular layer).

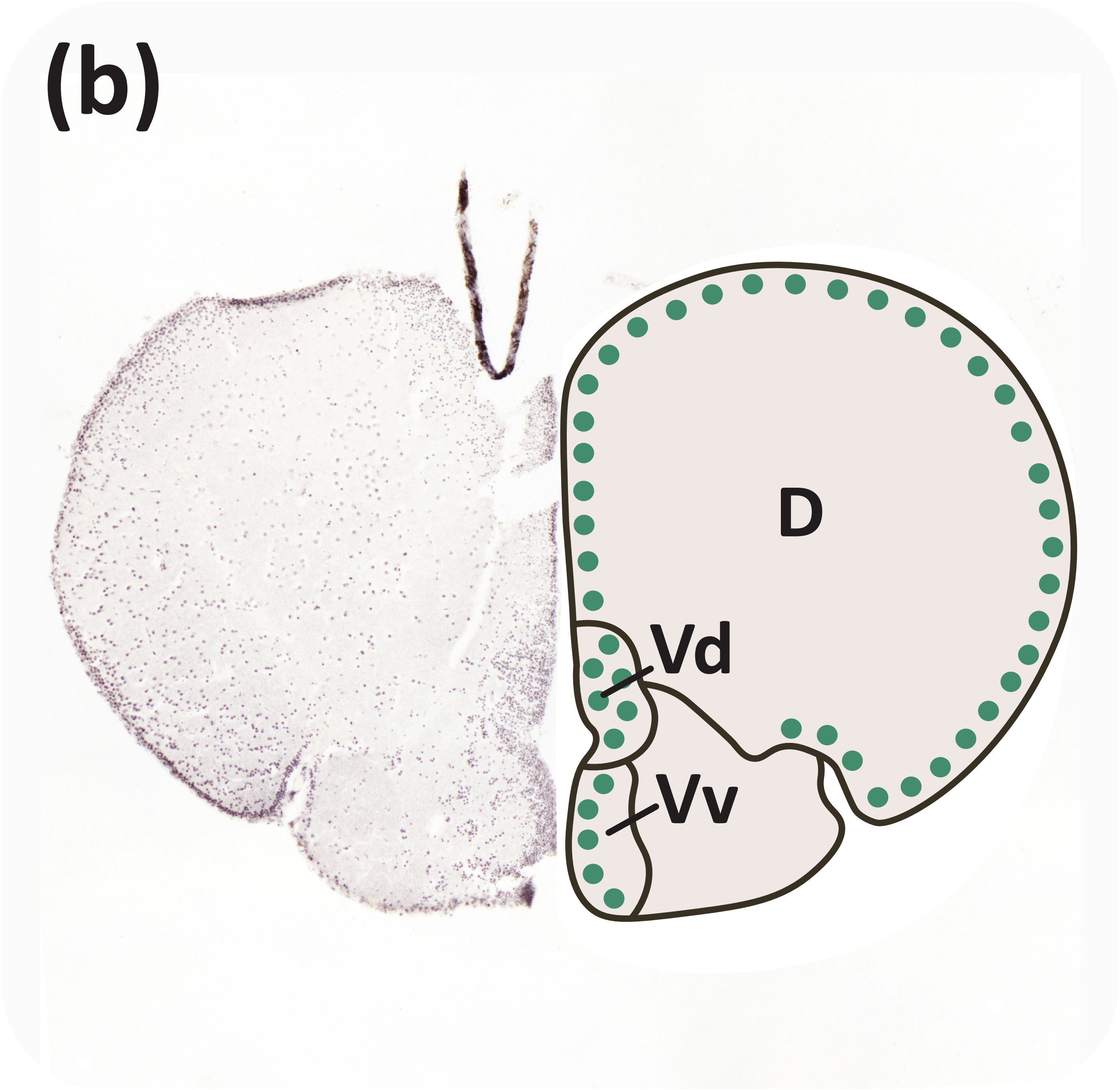

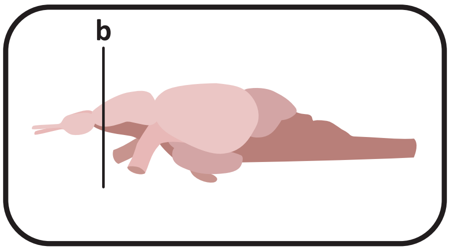

Abbreviations: D (dorsal telencephalon); Vd (ventral telencephalon, dorsal nucleus); Vv (ventral telencephalon, nucleus).

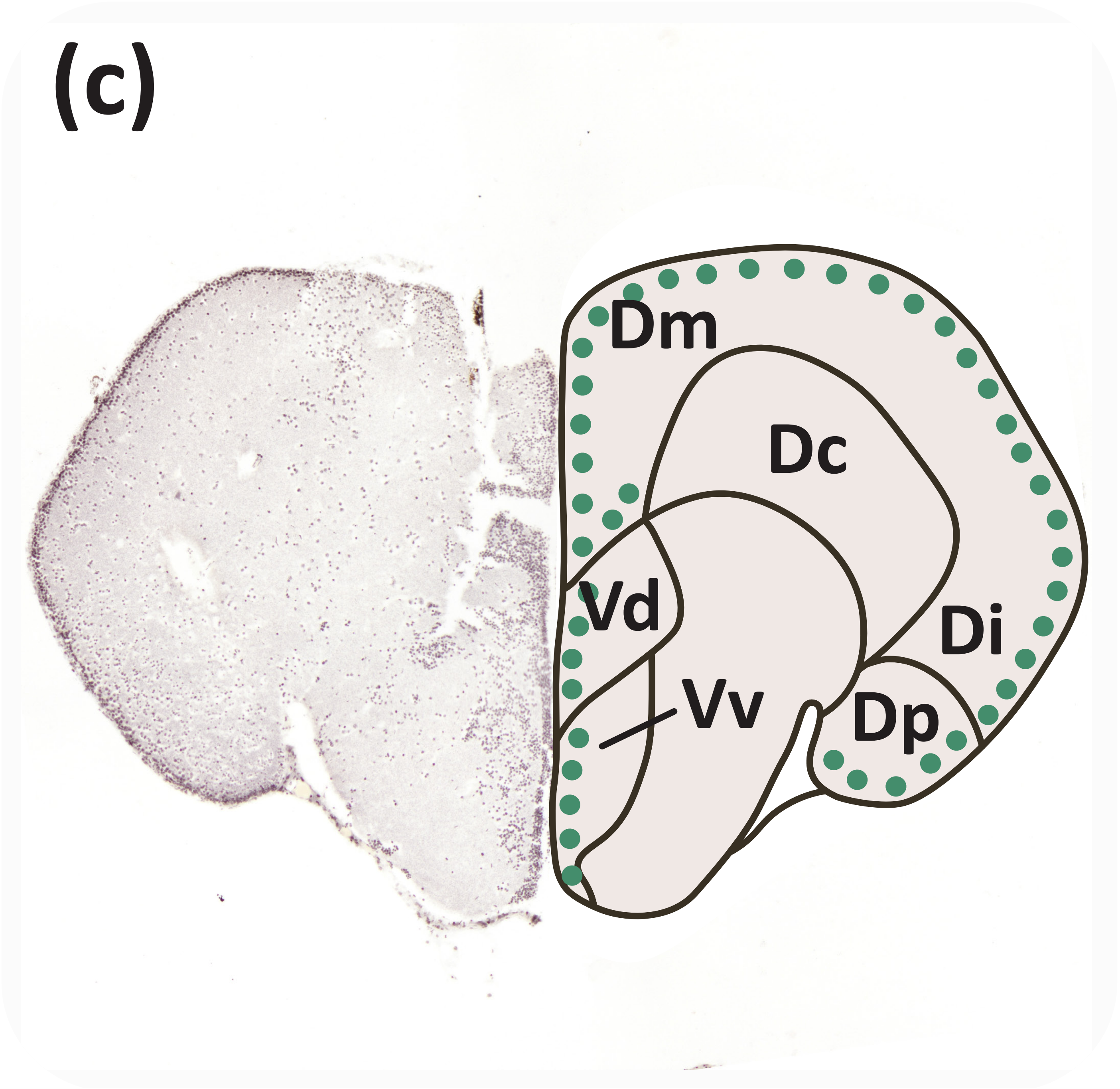

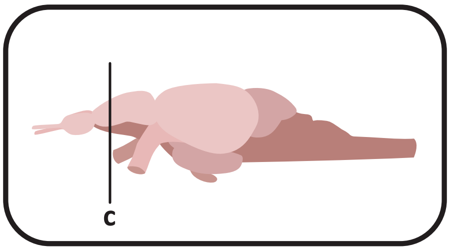

Abbreviations: Dc (dorsocentral telencephalon); Di (dorsolateral telencephalon); Dm (dorsomedial telencephalon); Dp (dorsoposterior telencephalon); Vd (ventral telencephalon, dorsal nucleus); Vv (ventral telencephalon, nucleus).

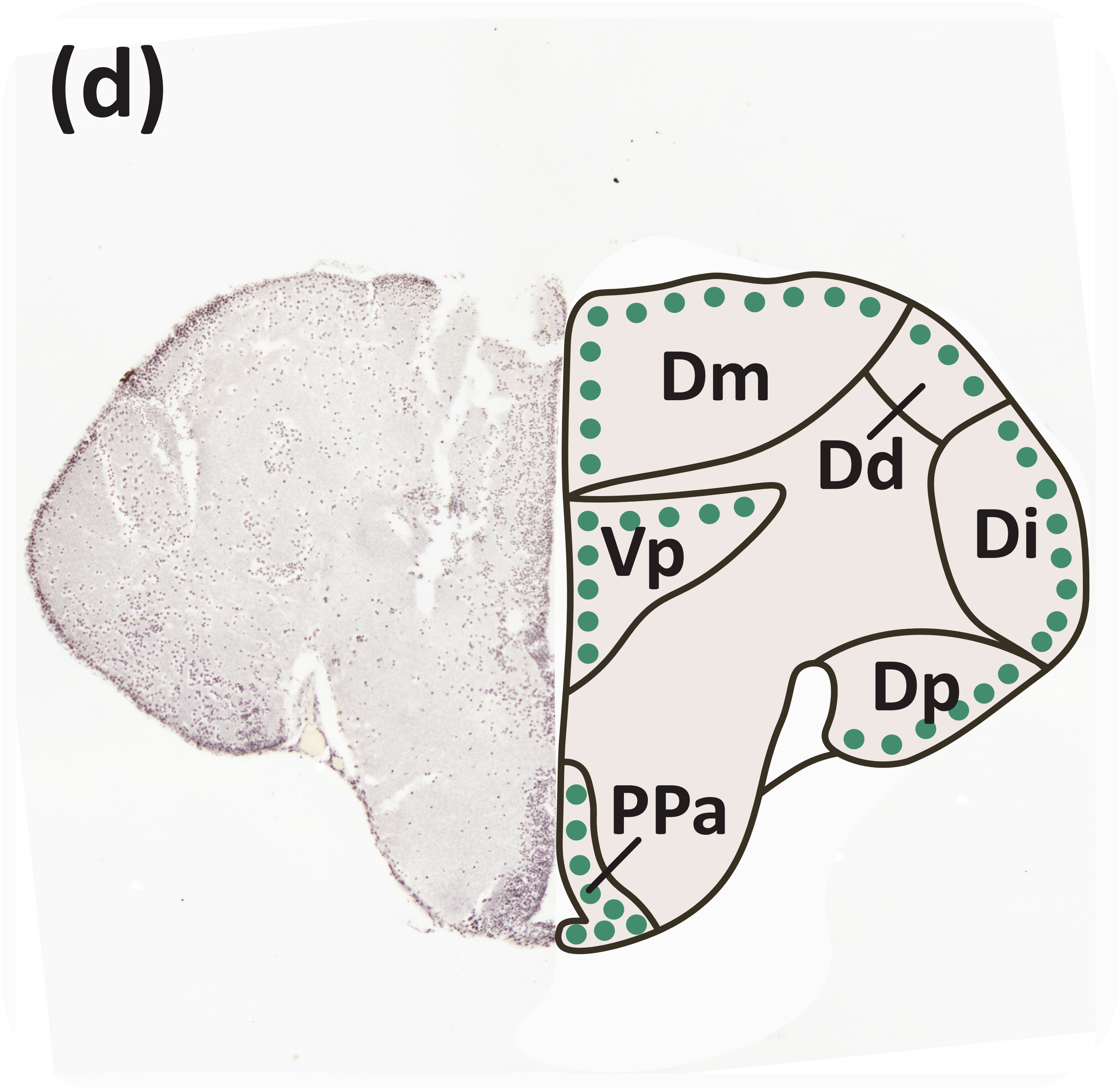

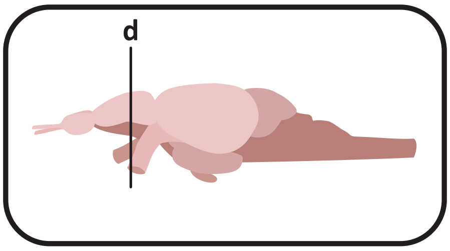

Abbreviations: Dd (dorsal region of dorsal telencephalon); Dc (dorsocentral telencephalon); Di (dorsolateral telencephalon); Dm (dorsomedial telencephalon); Dp (dorsoposterior telencephalon); PPa (parvocellular preoptic nucleus, anterior); Vp (ventral telencephalon, post-comissural nucleus).

(e)

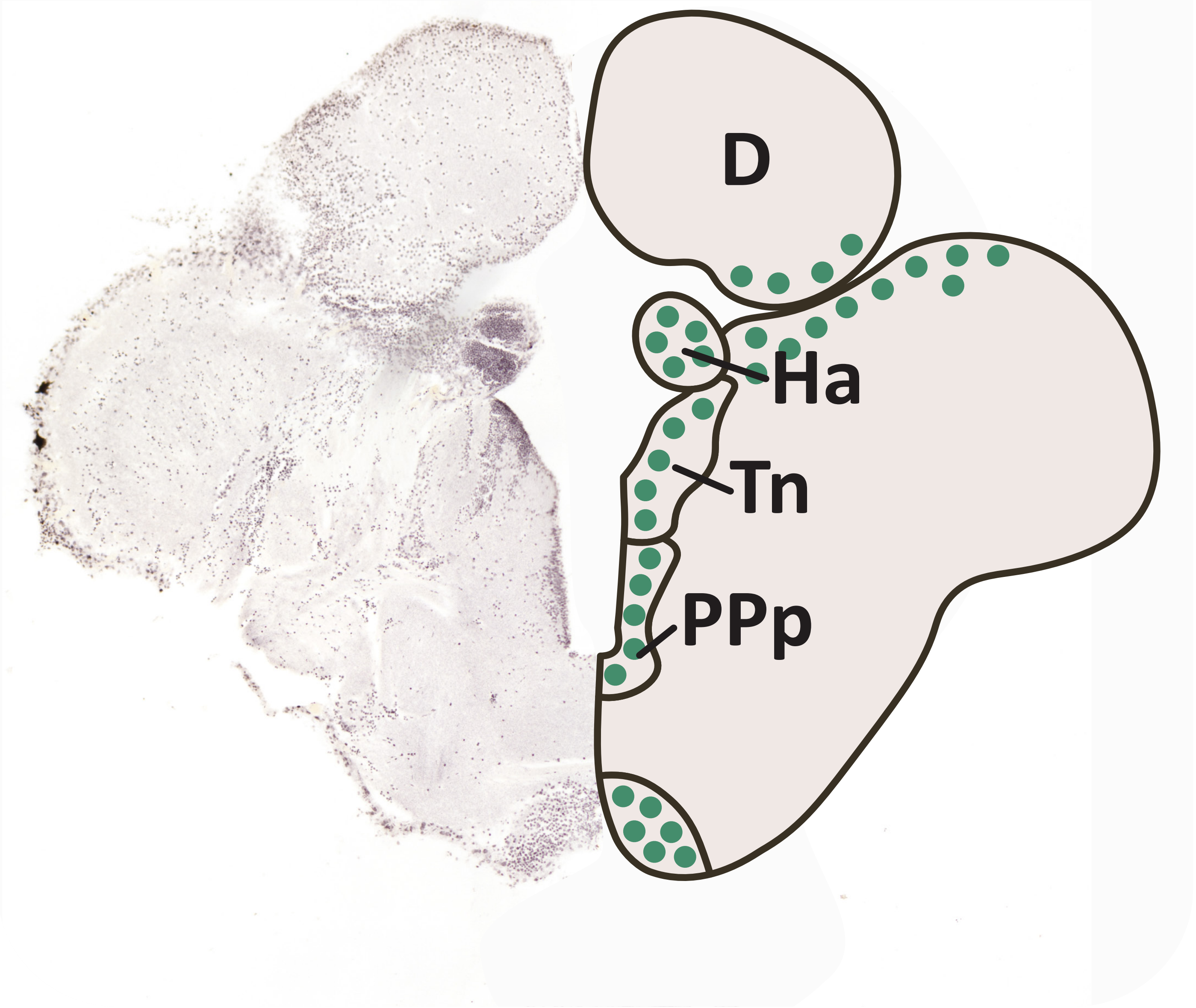

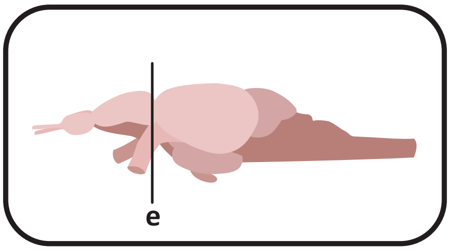

Abbreviations: D (dorsal telencephalon); Ha (habenular nucleus); PPp (parvocellular preoptic nucleus, posterior); Tn (thalamic nucleus).

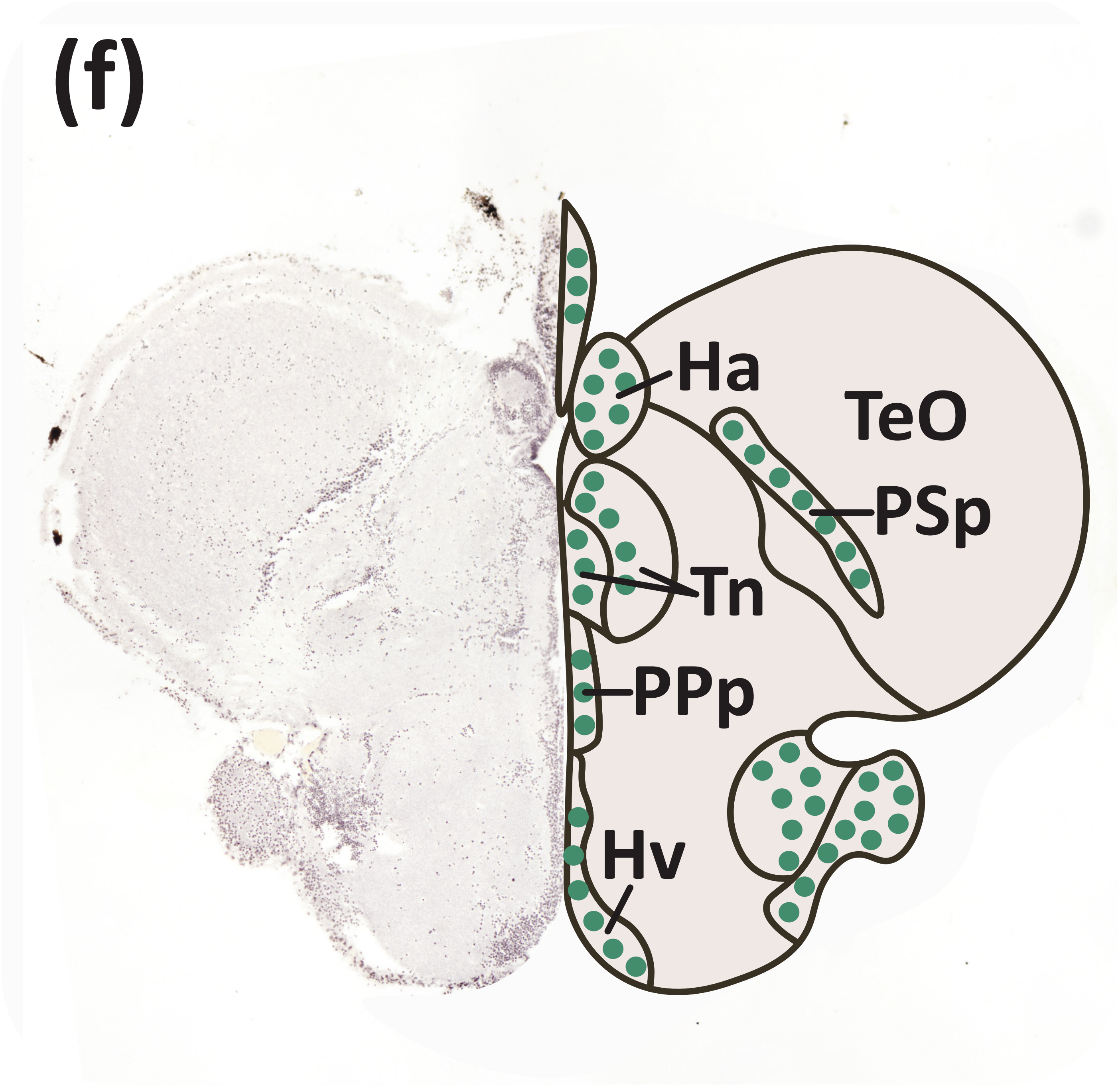

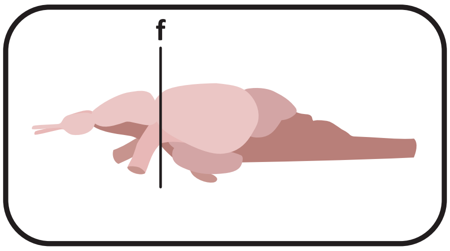

Abbreviations: Ha (habenular nucleus); Hv (periventricular hypothalamus, ventral); PSp (parvocellular superficial pretectal nucleus); TeO (optic tectum); Tn (thalamic nucleus).

(g)

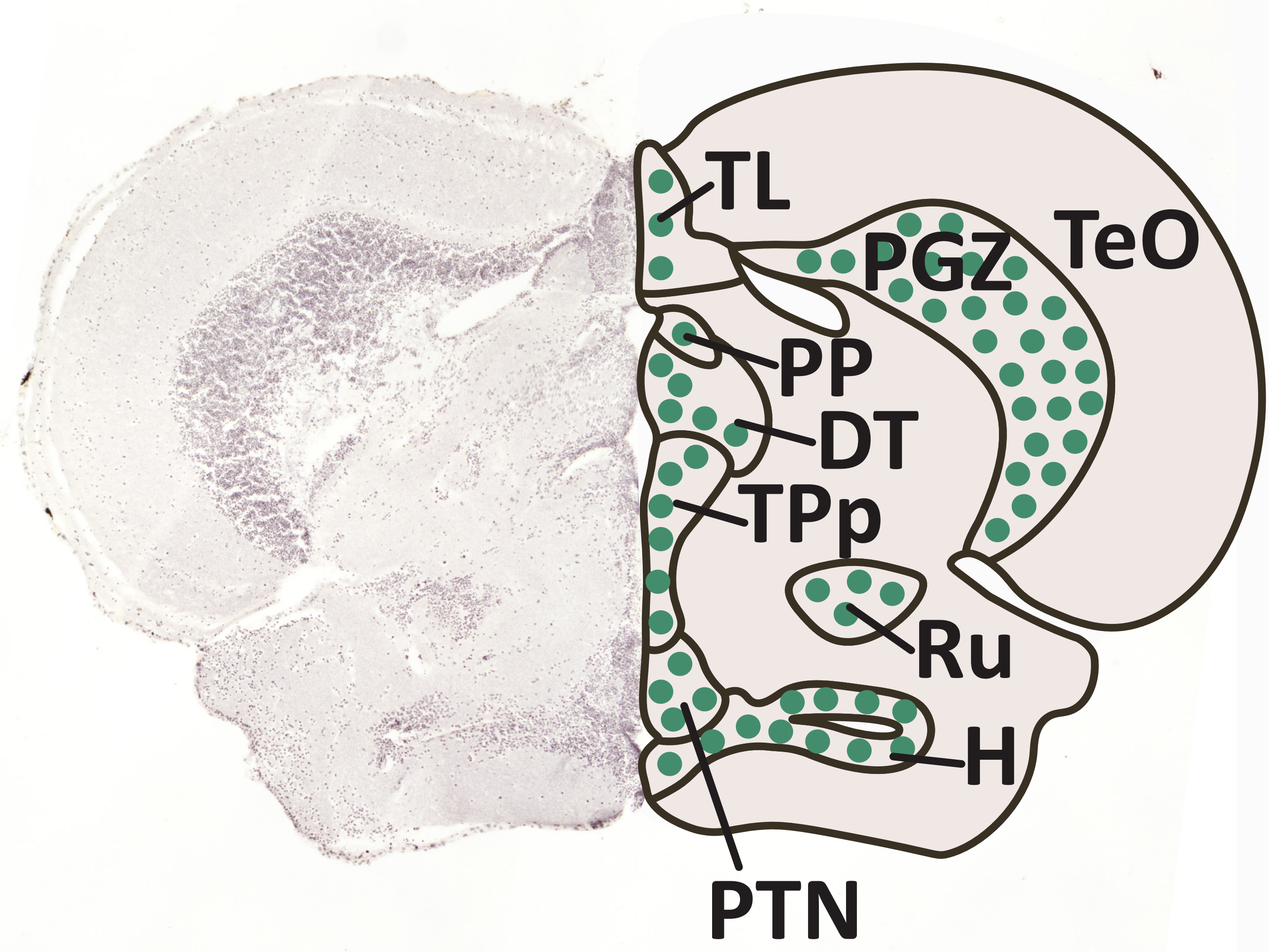

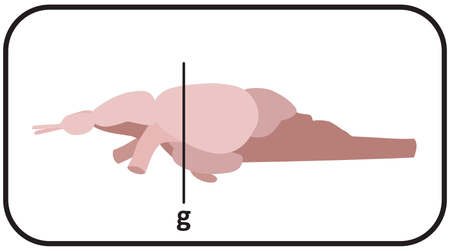

Abbreviations: DT (dorsal thalamus); H (hypothalamus); PGZ (periventricular gray zone of the TeO); PP (periventricular pretectal nucleus); PTN (posterior tuberal nucleus); RU (nucleus ruber); TeO (optic tectum); TL (torus longitudinalis); TPp (periventricular nucleus of posterior tuberculum).

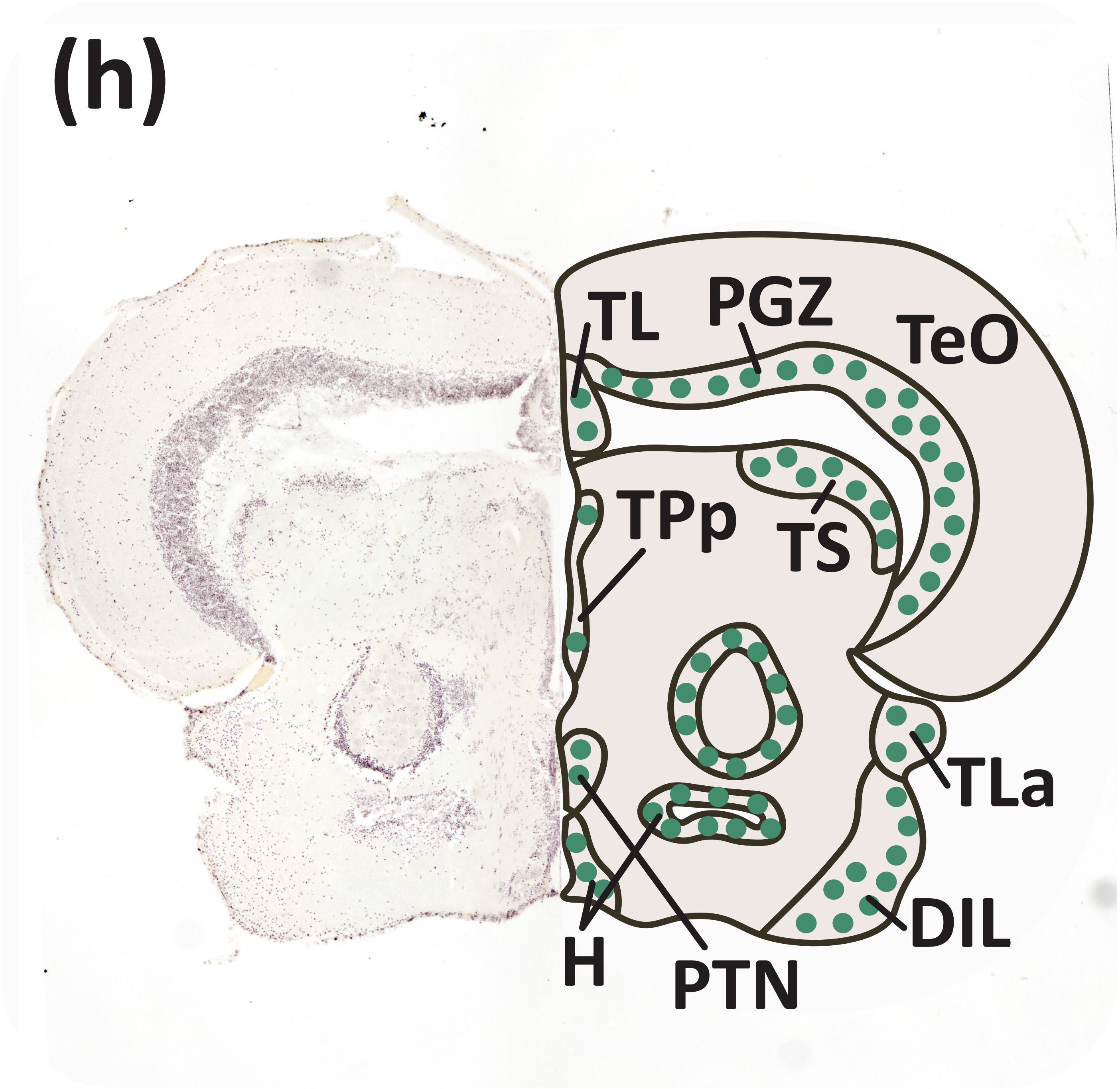

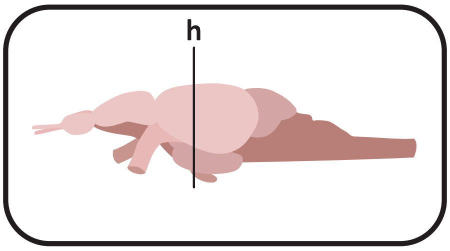

Abbreviations: DIL (diffuse nucleus of the inferior lobe); H (hypothalamus); PGZ (periventricular gray zone of the TeO); PTN (posterior tuberal nucleus); TeO (optic tectum); TLa (torus lateralis); TPp (periventricular nucleus of posterior tuberculum); TS (torus semicircularis).

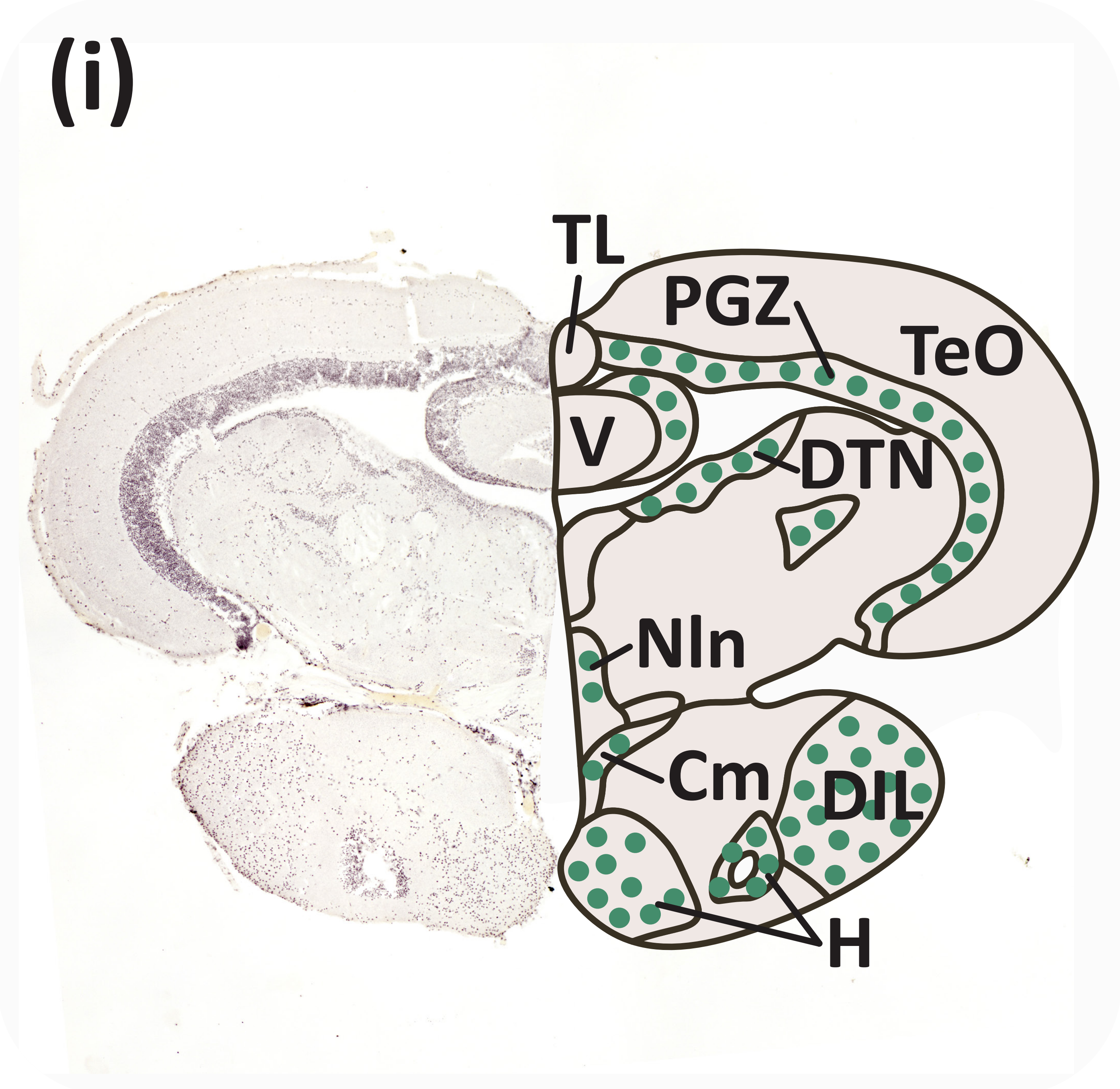

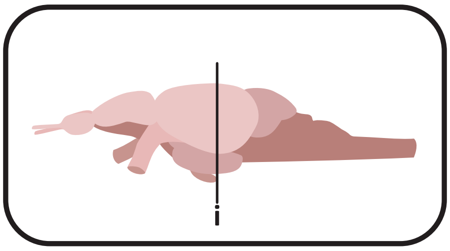

Abbreviations: Cm (corpus mamillare); DIL (diffuse nucleus of the inferior lobe); DT (dorsal thalamus); H (hypothalamus); NLn (nucleus interpeduncularis); PGZ (periventricular gray zone of the TeO); TeO (optic tectum); TL (torus longitudinalis); V (valvula cerebelli).

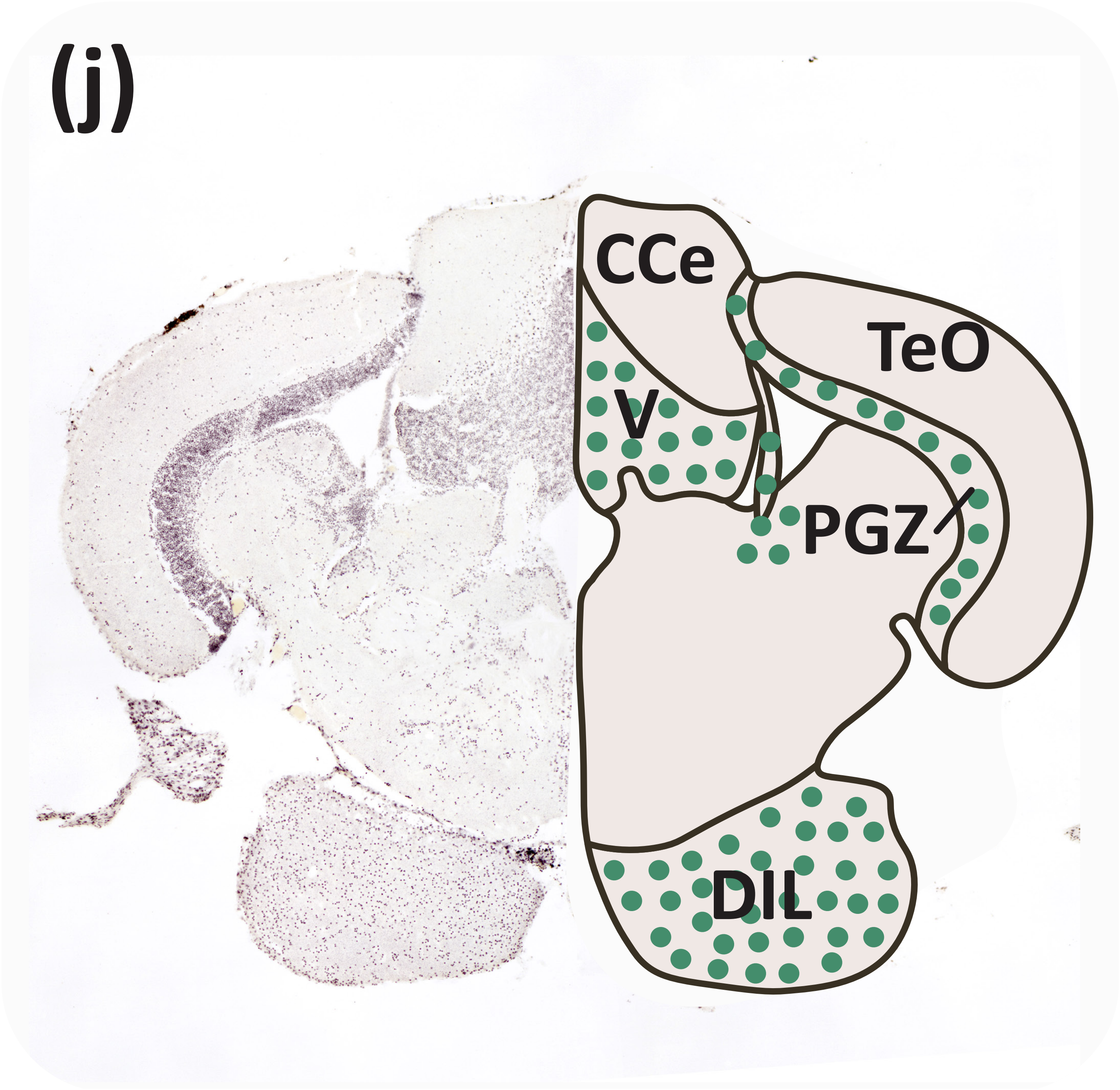

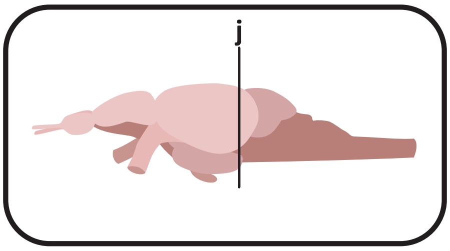

Abbreviations: Cce (corpus cerebelli); DIL (diffuse nucleus of the inferior lobe); PGZ (periventricular gray zone of the TeO); TeO (optic tectum); V (valvula cerebelli).

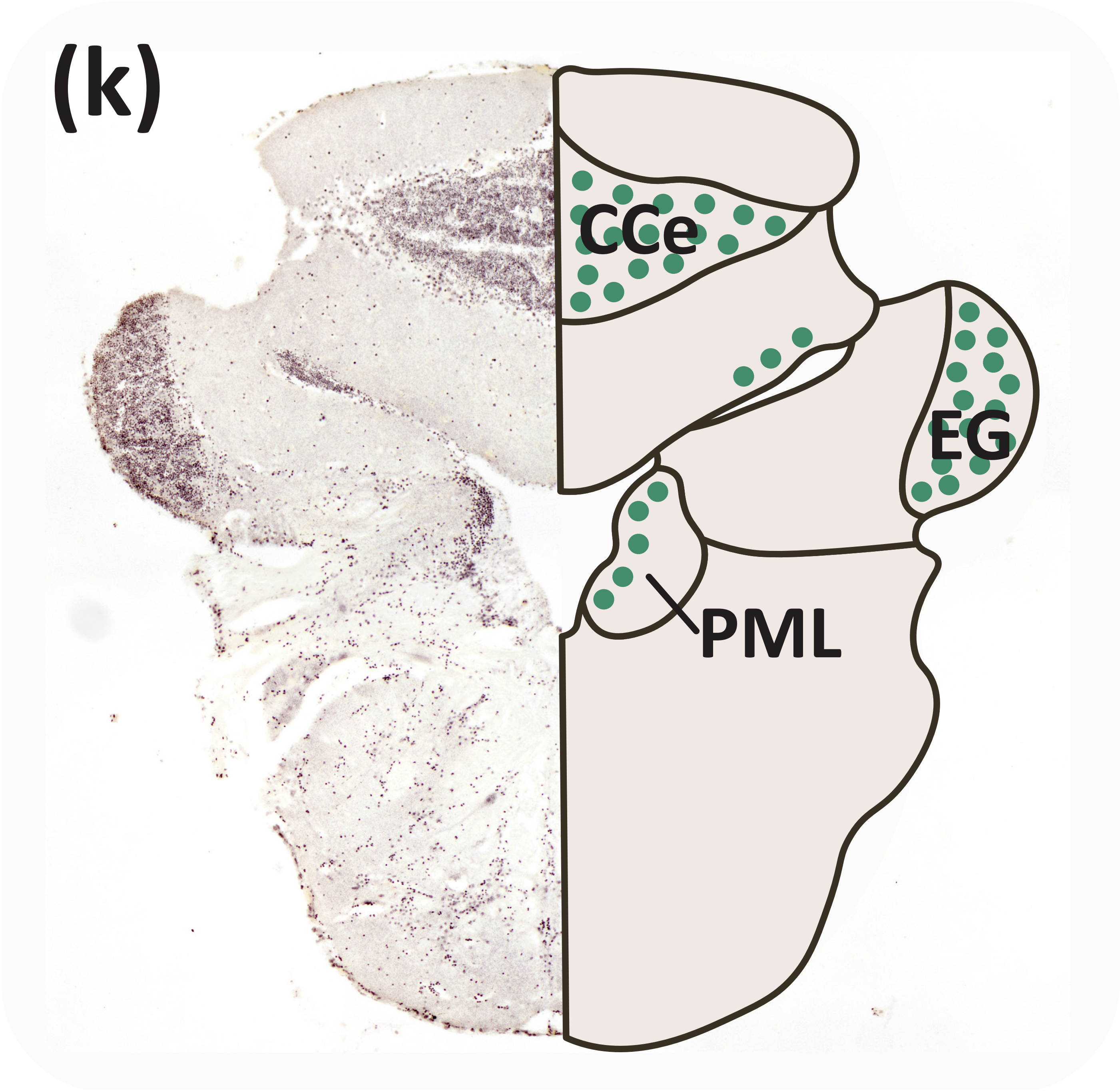

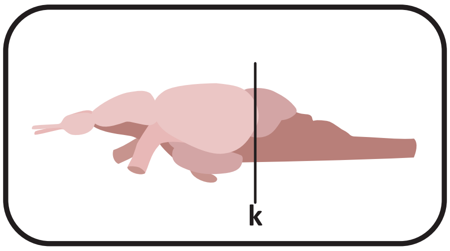

Abbreviations: Cce (corpus cerebelli); EG (eminentia granularis); PML (posterior mesencephalic lamina).

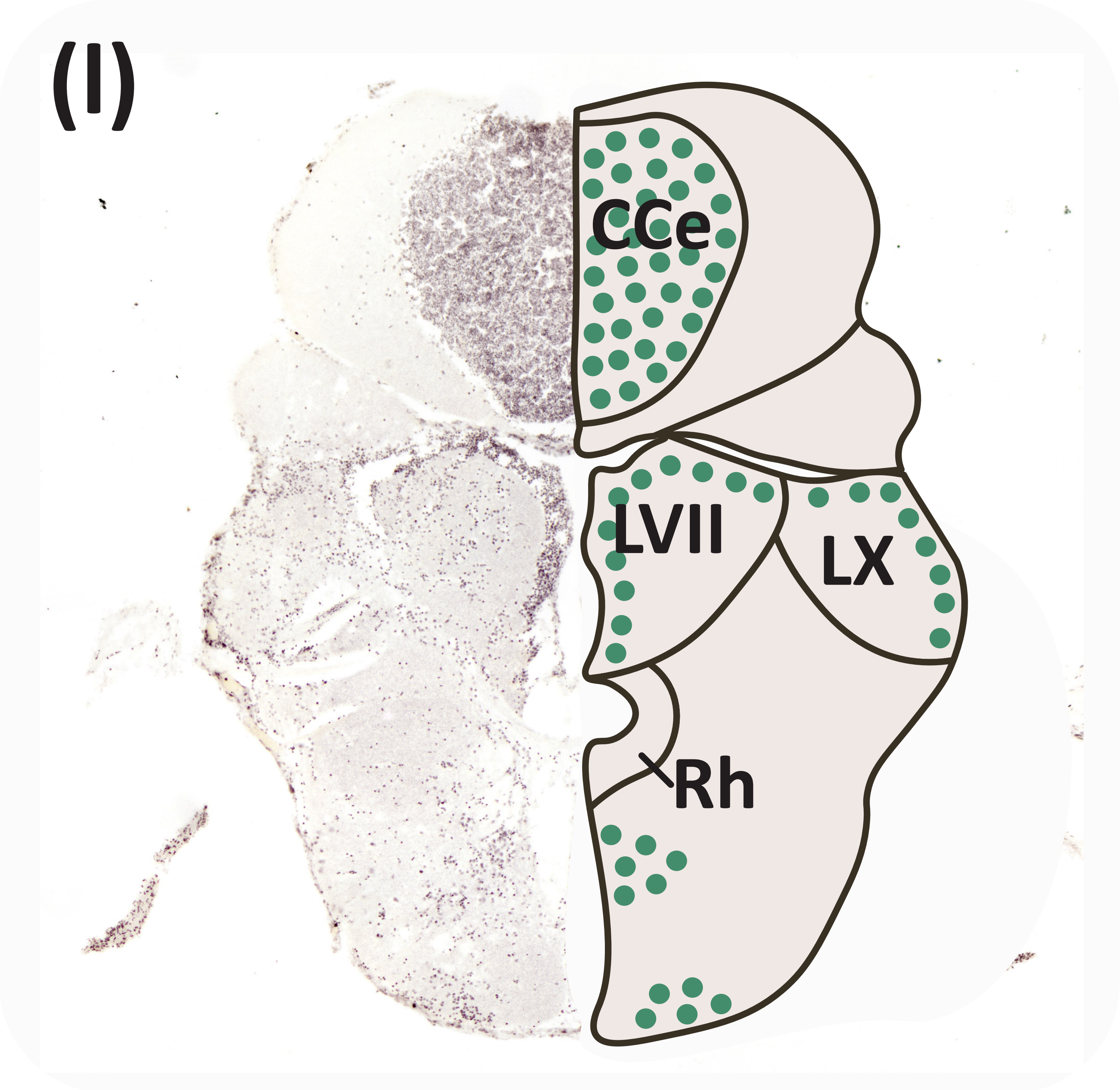

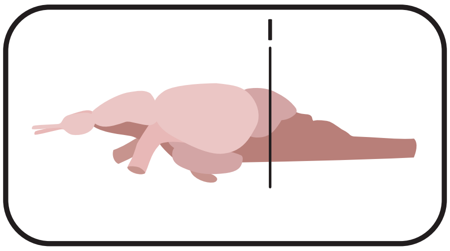

Abbreviations: Cce (corpus cerebelli); LVII (lobus facialis); LX (lobus vagus); Rh (ventricular zone of rhombencephalic ventricle).
